# Prior Exposure to Coxsackievirus A21 Does Not Mitigate Oncolytic Therapeutic Efficacy

**DOI:** 10.3390/cancers13174462

**Published:** 2021-09-04

**Authors:** William J. Burnett, David M. Burnett, Gennie Parkman, Andrew Ramstead, Nico Contreras, William Gravley, Sheri L. Holmen, Matthew A. Williams, Matthew W. VanBrocklin

**Affiliations:** 1 Department of Oncological Sciences, Huntsman Cancer Institute, University of Utah, Salt Lake City, UT 84112, USA; william.burnett@hci.utah.edu (W.J.B.); david.burnett@hci.utah.edu (D.M.B.); gennie.parkman@hci.utah.edu (G.P.); 2 Department of Pathology, Huntsman Cancer Institute, University of Utah, Salt Lake City, UT 84112, USA; andrew.ramstead@path.utah.edu (A.R.); nico.contreras@hci.utah.edu (N.C.); matthew.williams@path.utah.edu (M.A.W.); 3 School of Medicine, University of Nevada Las Vegas, Las Vegas, NV 89154, USA; gravley@unlv.nevada.edu; 4 Department of Surgery, Huntsman Cancer Institute, University of Utah, Salt Lake City, UT 84112, USA; sheri.holmen@hci.utah.edu

**Keywords:** oncolytic virus, coxsackievirus, CVA21, virotherapy, picornavirus, immunotherapy

## Abstract

**Simple Summary:**

Viruses are being explored as a treatment strategy for cancer by exploiting their ability to infect and kill malignant cells. Viruses also engage the immune system to promote the recognition and clearance of tumors from the body. A major concern with using viruses for therapy is anti-viral immunity, which could clear the virus before it can promote an anti-tumor response. We aim to determine the extent to which anti-viral immunity affects anti-tumor immunity. Our findings suggest that anti-viral immunity does not mitigate the anti-cancer effects of viral infection. Thus, virus therapy may be a valid treatment strategy, even in individuals that have pre-existing immunity to the virus.

**Abstract:**

Oncolytic viruses (OVs) are being developed as a type of immunotherapy and have demonstrated durable tumor responses and clinical efficacy. One such OV, Coxsackievirus A21 (CVA21), exhibited therapeutic efficacy in early phase clinical trials, demonstrating the ability to infect and kill cancer cells and stimulate anti-tumor immune responses. However, one of the major concerns in using this common cold virus as a therapeutic is the potential for innate and adaptive immune responses to mitigate the benefits of viral infection, particularly in individuals that have been exposed to coxsackievirus prior to treatment. In this study, we assess melanoma responses to CVA21 in the absence or presence of prior exposure to the virus. Melanomas were transplanted into naïve or CVA21-immunized C57BL6 mice and the mice were treated with intratumoral (IT) CVA21. We find that prior exposure to CVA21 does not dramatically affect tumor responses, nor does it alter overall survival. Our results suggest that prior exposure to coxsackievirus is not a critical determinant of patient selection for IT CVA21 interventions.

## 1. Introduction

Melanoma is a cancer of the skin that has been increasing in incidence over the past 50 years. It is estimated that there will be 106,110 new cases of melanoma in the United States in 2021 [1]. Though relatively rare, melanoma is the deadliest form of skin cancer, accounting for more than 62% of skin cancer deaths (~7180 deaths expected in 2021), with a 5 year survival rate of only 27% in advanced stage cases [1]. Approximately 1 in 27 males and 1 in 40 females risk developing invasive melanoma in their lifetime. Melanoma mortality is largely attributed to its highly metastatic nature, with secondary lesions often spreading to the lungs, liver, skin, brain, and bone [2].

Despite these sobering statistics, there has been an average annual percent decrease in melanoma mortality of about 5.7% since 2014, most likely attributable to the advent of immune checkpoint blockade (ICB) interventions [1,3,4,5,6,7]. These biologics act to inhibit negative regulatory signals from the tumor cell to the T-cell, thereby allowing the T-cells to recognize the tumor as a foreign entity for targeted elimination. When driven by ICB interventions, tumor responses have proven to be robust and durable [1,7]. Though powerful, ICB has only benefited a subset of melanoma patients. Mechanisms of resistance to these therapies are being identified in model systems and clinical studies, and include a lack of infiltrating CD8+ T-cells or the presence of exhausted CD8+ T-cells within the tumor, which appear to be crucial for strong responses [8,9,10,11]. A major focus in the field is to identify treatment combinations to compliment these checkpoint inhibitors to expand tumor responses.

Oncolytic viruses (OV) have had a resurgence as prospective cancer therapeutics with the advent of immune checkpoint inhibitors. The ability of OVs to be delivered intratumorally and/or systemically, to recruit immune cells to the site of infection, to replicate within and kill tumor cells, and to drive interferon responses while having relatively low toxicities make them attractive candidates for combination with checkpoint blockade [12,13]. OVs are being evaluated in dozens of clinical trials in many different cancer types, with principal success in melanoma [14].

Coxsackievirus A21 (CVA21/CAVATAK) is an emerging therapeutic agent for the treatment of advanced melanoma. It is a wild-type, positive sense, single stranded, non-enveloped picornavirus that commonly causes upper respiratory tract infections. CVA21 exploits its primary required receptor ICAM-1 (a well-characterized glycoprotein on the cell surface) for cell entry [15,16,17]. Importantly, ICAM-1 is highly expressed in melanoma but not in most other tissues, accounting for CVA21-melanoma tropism [18]. CVA21 is capable of infecting normal cells that express ICAM-1, however, these cells can prevent the spread of infection through innate antiviral defenses [19,20]. Melanoma and other malignant cells often exhibit impaired antiviral defenses and those expressing high levels of ICAM-1 can be efficiently infected and lysed by the virus, resulting in tumor destruction accompanied by innate and adaptive immune responses [12,18,21,22]. Though incompletely understood, oncolysis results in release of tumor antigens that can facilitate anti-tumor adaptive immunity as well [12,22]. Thus, in the course of an anti-viral response, anti-tumor immunity is developed. Additionally, high levels of ICAM-1 in metastases offer the potential for treatment of metastatic disease [23,24]. CVA21/CAVATAK has been proven to be safe and has demonstrated anti-tumor responses in a number of different cancers [25,26] Addressing concerns of the safety of this virus as a therapeutic, the CAVATAK in Late Stage Melanoma (VLA-007 CALM) Phase-II clinical trial demonstrated that CAVATAK was well tolerated in patients, with high response rates accompanied by no grade 3 or 4 adverse events [26]. CVA21 has the potential to enhance tumor infiltration and immune activation by leveraging natural immunity to the viral infection.

Nevertheless, one of the chief concerns with CVA21 is that a subset of people will have neutralizing antibodies developed from natural exposure to coxsackievirus-family members throughout their lifetime [27]. This concern is shared for a number of different OVs. It is speculated that neutralizing antibodies would work to immobilize the virus before it could have clinical benefit [28]. It is for this reason that patients below a certain neutralizing antibody titer were selected to participate in the CALM study [26]. Efforts are being made to circumvent or to directly counteract OV neutralization to enhance their anti-tumor effect [27,29]. Presumably, tumor responses to CVA21 infection would be much less impressive in patients who had prior exposure to the virus, although this has not been formerly tested.

We aimed to assess the extent to which prior exposure to CVA21 affects its efficacy as a therapeutic intervention. Using a syngeneic murine model of melanoma, we evaluated CVA21 in naïve and CVA21-immunized mice. We find that IT delivery of CVA21 promotes robust tumor regression in a high percentage of cases, yet the response is not significantly affected by prior exposure to the virus. Our findings indicate that, at least for CVA21, prior exposure to coxsackievirus and generation of CVA21-targeted antibodies may not be a critical determinant of patient selection for CVA21 interventions.

## 2. Materials and Methods

### 2.1. Cell Culture and Cell Lines

Normal human epithelial melanocytes (NHEM) were purchased from Invitrogen (Waltham, MA; USA). We have previously described the following human melanoma cell lines: A375, C32, C8161R, CACL, LOX IMVI, M14-MEL, MALME-3M, SK-MEL-28, SK-MEL-5, SK-MEL-2, SK-MEL-103, SK-MEL-147 UACC-62, UACC-257 [30]. CHL-1 and HeLa-H1 cells were purchased from American Type Culture Collection (ATCC, Manassas, VA, USA). Yale University Mouse Melanoma 2.1 (YUMM 2.1) cells were gifted by Dr. Martin McMahon [31]. Human melanoma cell lines were cultured in RPMI 1640 supplemented with 7% fetal bovine serum (FBS) and gentamicin. YUMM 2.1 cells were cultured in DMEM/F12 supplemented with 10% FBS, non-essential amino acids (NEAA), and gentamicin. NHEM cells were cultured in 254 media supplemented with human melanoma growth supplement, 10% FBS, NEAA, and gentamicin. Cells were cultured in humidified incubators at 37 °C with 5% CO_2_.

### 2.2. Western Blotting

Cell lysates were prepared by washing cells once in phosphate-buffered saline (PBS) followed by the addition of 0.5 mL of 0.25% Trypsin to remove cells from the tissue culture plates. Cells were collected in RPMI media (7% FBS; gentamicin) washed in PBS, and re-suspended in cold RIPA buffer with protease inhibitors and EDTA for protein extraction. Cells were lysed by agitation on a rotating platform at 4 °C for 20 min and clarified at 17,000× *g* for 10 min at 4 °C. Protein was quantified using a bicinchoninic acid (BCA) protein assay (Pierce; Waltham, MA; USA; 23225) according to manufacturer’s instructions. Following quantification, 10 µg of protein were diluted in 4× lithium dodecyl sulfate (LDS) buffer (Life Technologies; Carlsbad, CA; USA; NP0008) with dithiothreitol, denatured at 95 °C for 5 min, and loaded on to 4–12% Bis-Tris gels (Life Technologies; Carlsbad, CA; USA;NP0321BOX/NP0323BOX). Gels were run at 200 volts for 40 min. Proteins were transferred in NuPAGE transfer buffer (Thermo Fisher; Waltham, MA; USA; NP0006) to a nitrocellulose membrane (Bio-Rad; Hercules, CA; USA; 162-0232) at 90 volts for 90 min and blocked in 5% non-fat dry milk in 0.05% TBS-T for 30 min at RT. Membranes were washed several times in 0.05% TBS-T, and probed with primary antibodies diluted in 5% BSA with agitation at 4 °C. The membrane was washed several times in 0.05% TBS-T. Anti-mouse (Cell Signaling Technologies; Danvers, MA; USA; 7076S) and anti-rabbit (Cell Signaling Technologies; Danvers, MA; USA; 7074S) secondary antibodies were diluted 1:1000 in 0.05% TBS-T and used to probe the membrane for 1–2 h with agitation at 4 °C. The membrane was then washed several times in 0.05% TBS-T and proteins were detected using enhanced chemiluminescence (ECL) reagent (Cytvia; Marlborough, MA; USA; RPN2106). Antibodies used include ICAM-1 (Cell Signaling Technologies; Danvers, MA; USA; Rabbit; 4915), HA (Cell Signaling Technologies; Danvers, MA; USA; Rabbit; 3274), and GAPDH (Millipore; Burlington, MA; USA; Mouse; MAB374).

### 2.3. Reverse-Transcription Polymerase Chain Reaction (RT-PCR)

RT-PCR to detect CVA21 was carried out in two steps. Reverse transcription was performed on RNA samples using the ProtoScript II first strand cDNA synthesis kit (New England BioLabs; Ipswich, MA; USA E6560S) with a CVA21-specific reverse primer (Rev 5′-gagtcgagccatcggcggtac-3′). The reaction was carried out at 25 °C for 5 min, then 42 °C for 1 h followed by inactivation of the enzyme at 80 °C for 5 min. Once cDNAs were generated for each sample, CVA21 amplicons from the 3′ end of the viral genome in the 3D^pol^ (408 bp) were generated by polymerase chain reactions (PCR) using EconoTaq PLUS Green 2X Master Mix (Lucigen; Middleton, WI; USA; 30033-1) and CVA21-specific primers (Fwd 5′-attgcctatggtgatgacgtg-3′; Rev 5′-gagtcgagccatcggcggtac-3′). The PCR was run with an initial 95 °C denaturing step for 10 min, followed by 21 cycles of denaturing at 95 °C for 20 s, annealing at 55 °C for 20 s, and elongation at 68 °C for 30 s. The PCR was concluded with a final elongation step of 72 °C for 5 min prior to cool down to 4 °C. Amplified DNA was detected by running 25 µL of the PCR product on a 1% agarose gel stained with ethidium bromide and imaged with a UV illuminator.

### 2.4. CVA21 Propagation, Purification, and Titration

CVA21 was propagated in HeLa-H1 cells (ATCC; Manassas, VA; USA). A 10 cm dish of HeLa-H1 cells was infected at 90–100% confluence with 1 × 10^6^ IFU (MOI 0.1) for 16–24 h, whereupon cells were observed for signs of cytopathological effect (CPE). Upon confirmation of >50% CPE, cells were scraped into the culture media, collected into conical tubes, and flash frozen using a methanol-dry ice bath. Virus was released from infected cells by thawing in a 47 °C water bath followed by rigorous vortexing for 30 s. Freeze–thaw cycles were carried out three times on samples before being cleared at 5000 rpm for 10 min at 4 °C. Viral supernatants were then 0.8 µm filtered into clean tubes. Viral stocks were stored at −80 °C in aliquots.

To generate purified viral stocks, HeLa-H1 cells in suspension culture were allowed to grow between 5 × 10^5^ and 1.5 × 10^6^ cells/mL in 800 mL of S-MEM media (10% FBS, Non-essential amino acids (NEAA), Pleuronic-F68, L-glutamine, gentamicin). Cells were then pelleted at 500× *g* for 10 min, re-suspended in 45 mL of media, and infected with 35 mL of propagated CVA21 stock. The infection was carried out at 37 °C for 1 h with shaking at 70 rpm. A total of 80 mL of fresh S-MEM was then added to the flask and the infection proceeded at 37 °C with shaking at 120 rpm until widespread (>50%) CPE was observed. The cell suspension was freeze–thawed and cleared as previously described and aliquoted equally into 25 × 89 mm SW 28 ultracentrifuge tubes (Beckman Coulter; Pasadena, CA; USA; 326823). Then, 1.5 mL of a buffered 30% sucrose solution (20mM Tris, 1M NaCl) was added to the bottom of each tube to create a sucrose cushion. Tubes were weighed in rotor buckets and balanced with PBS. CVA21 cultures were ultracentrifuged at 25,000 rpm for 4 h at 4 °C. Supernatant was decanted and the virus pellets were suspended in 100 µL of PBS, covered, and placed at 4 °C for 1 h to allow the pellets to dissolve. The pellets were then combined, suspended in 4 mL of PBS, and stored at −80 °C in 50 µL aliquots.

To determine viral titer, YUMM 2.1 ICAM-1 cells were seeded in two 6-well cell culture dishes at 50–60% confluence. A ten-fold serial dilution of the viral stocks was prepared in Opti-MEM media and cells were infected with 500 µL of each dilution for 2 h at 37 °C. Following the infection, cells were washed in PBS to remove unincorporated virus and then incubated in 1 mL of normal culture media (DMEM/F12; 10% FBS, NEAA, gentamicin) for 72 h at 37 °C. RNA was then purified from the cells by TRIzol (Thermo Fisher Scientific; Waltham, MA; USA;15596018) extraction according to manufacturer’s instructions. Cells were washed in PBS and 400 µL of TRIzol reagent was added to each well. Wells were rinsed vigorously with the TRIzol and cells were collected into tubes and incubated at RT for 5 min. Then, 80 µL of chloroform was then added and samples incubated for an additional 3 min. Separation of the aqueous, inorganic, and organic layers was carried out by centrifugation at 12,000× *g* for 15 min at 4 °C. The aqueous phase was then transferred to a tube containing 15 µg of GlycoBlue (Thermo Fisher Scientific; Waltham, MA; USA AM9515). RNA was precipitated with 200 µL of isopropanol and incubated for 10 min at room temperature prior to being cleared at 12,000× *g* for 10 min at 4 °C. The supernatant was discarded and the pellet was re-suspended in 75% ethanol and vortexed prior to centrifugation at 7500× *g* for 5 min at 4 °C. The supernatant was removed and the pellet was allowed to air-dry at room temperature for 10 min. Samples were re-suspended in 20 µL of RNase-free water and heated at 55 °C for 15 min. RT-PCR was then performed to detect CVA21 in the extracted RNA. RNAs were stored at −80 °C.

### 2.5. Flow Cytometry

Cell death assays were performed using the Attune Nxt Acoustic Focusing Cytometer (Life Technologies; Carlsbad, CA; USA). Cells were seeded in 6-well plates at a density of 1 × 10^6^ cells per well. The following day cells were infected with CVA21 at an MOI of 0, 0.1, 1.0, or 10 in phenol red-free RPMI (7% FBS; gentamicin) for 1 h. Cells were then washed with PBS, re-fed normal media, and incubated for 24 to 48 h. At the experimental endpoint media was collected and combined with a PBS wash for each sample. Following treatment with 0.25% trypsin, adherent cells were re-suspended in the media/PBS mixture from each well. Cells were pelleted by centrifugation at 500× *g* for 10 min at 4 °C and re-suspended in 1 ml of 0.2 µm SYTOX Green (Invitrogen; Waltham, MA; USA; S7020) reagent diluted 1:1000 in PBS from a 5 mM stock. Cells were stained in the dark at room temperature (RT) for 20 min prior to being stored on ice and run on the Attune (Life Technologies; Carlsbad, CA; USA). Cells were detected using a blue laser (488 nm excitation BL1 530/30 bandpass filter) and analyzed for uptake of the dye indicative of cell death. Cell death was quantitated by averaging the percentage of cell death in three replicates. Error bars were generated based on the calculated standard deviation of the replicates.

Tumor profiling was performed using the BD LSRFortessa flow cytometer. Mouse immunization, tumor initiation, and tumor treatments were performed as described. At the experimental endpoint, mice were euthanized and tumors were collected into pre-weighed tubes containing 5 mL of serum-free RPMI media. Samples were then weighed again to determine the mass of each tumor collected. Tumors were dissociated in the RPMI using scissors and forceps and by grinding the tumors between frosted glass slides. A total of 250 µL of 20× Collagenase IV/DNAse was then added and dissociated tumor samples were incubated with gentle shaking at 37 °C for 45 min. Following the collagenase digestion, samples were 40 µm filtered followed by an additional wash of 5 mL of serum-free RPMI into the same collection tube. Cells were collected by centrifugation at 1500 rpm for 5 min at 4 °C and decanted prior to resuspension in 1 mL of ACK (Ammonium-Chloride-Potassium; pH 7.22) buffer. Cells were again cleared by centrifugation as before and resuspended in 300 µL of RPMI (2% FBS) and 3 wells/mouse were plated in round-bottomed 96-well plates at 100 µl/well. Cells were pelleted in the plate at 2000 rpm for 1 min and stained using three different panels, including myeloid (F480 (APC), CD45 (AF488), CD86 (BV421), Ly6C (BV510), Live/Dead Aqua (BV605), CD80 (BV650), CD11b (BV711), MHCII (IA/IE) (BV786), B220 (PE), CD11c (PE-CF594), CD8a (PECy7), Fc Block), lymphoid (CD44 (APCe780), CD45 (AF488), CD127 (PerCP Cy5.5), Live/Dead Aqua (BV510), PD-1 (BV605), CD4 (BV650), KLRG1 (BV711), CD8a (BV786), CXCR6 (PE), CD62L (PE-CF594)), and transcription Factor panels (FoxP3 (APC), BCL6 (APC Cy7), CD45 (AF488), GrzB (BV421), Live/Dead Aqua (BV510), Tbet (BV605), CD4 (BV650), CD8a (BV786), TCF1 (PE)).

Staining for myeloid and lymphoid panels was carried out on ice in the dark for 30 min and clarified at 2000 rpm for 1 min. Stained samples were then washed three times in 1× PBS and clarified under the same conditions. Cells were stained again with live/dead stain (1:1000 in PBS) for 10 min in the dark at RT. Following live/dead staining, samples were washed three times in FACS buffer and clarified as before. Myeloid and Lymphoid panels were then fixed in Cytofix/Cytoperm for 5 min at RT in the dark, washed three times in FACS buffer, and resuspended in 200 µL of FACS buffer prior to overnight incubation at 4 °C in the dark.

Cells used for the transcription factor panel were permeabilized in FOXP3 perm buffer (eBiosciences; Waltham, MA; USA) and washed in FOXP3 perm wash (eBiosciences; Waltham, MA; USA). They were fixed in FOXP3 Fix buffer for 10 min at RT in the dark followed by additional washes in FOXP3 perm buffer. Antibodies for the transcription factor panel were suspended in FOXP3 perm buffer and cells were stained on ice for 30 min. Cells were then washed once in perm buffer and twice in FACS buffer, followed by a final resuspension in FACS buffer and incubated at 4 °C overnight in the dark.

Data were analyzed using a two-way ANOVA test with a Tukey post-test. *p* values < 0.05 were considered significant (*p* < 0.05 *, < 0.01 **, < 0.001 ***, < 0.0001 ****).

### 2.6. CVA21 Enzyme Linked Immunosorbent Assay (ELISA)

Serum samples were collected from naïve and immunized mice as described previously. A 96-well ELISA plate (VWR; Radnor, PA; USA; 3590) was coated with CVA21 by incubating each well in 2 × 10^5^ IFU of CVA21 in 100 µL of PBS for 1 h at RT. Coated plates were stored overnight at 4 °C. CVA21 was discarded and wells were incubated in 250 µL of blocking buffer (Thermo Fisher; TBS Buffer 28358, 0.5% Tween, 0.5% BSA) for 1 h at RT. Blocking buffer was then discarded and mouse serum samples were diluted 1:50, 1:500, and 1:5000 in blocking buffer and incubated in the antigen coated wells for 1 h at RT. The plate was then washed three times in 250 µL of wash buffer (Thermo Fisher; TBS Buffer 28358, 0.5% Tween) and incubated in HRP-conjugated goat anti-mouse IgG secondary antibody (Thermo Fisher; 31430) diluted 1:5000 in blocking buffer. Wells were incubated in 100 µL of secondary antibody for an hour at RT. Following four additional wash steps, 50 µL of TMB reagent (Thermo Fisher; 34028) was added to each well. After 15–30 min, 50 µL of stop solution (Thermo Fisher; SS04) was added to the plate and the absorbance at 450 nm was quantified using a plate reader.

### 2.7. Mice and Injectables

C57BL6 (BL6) mice were obtained from the Charles River Laboratories (Raleigh, NC; USA). Immunizations were performed 4 weeks prior to tumor initiation by IP injection of 6 week old mice with a mixture of 2 × 10^7^ IFU of CVA21 in an equal volume of Magic Mouse adjuvant (Creative Diagnostics; Shirley, NY; USA; CDN-A001). This adjuvant stimulates an immune response through CpG DNA oligodeoxynucleotides that engage Toll-like receptors. The purpose of the adjuvant is to stimulate anti-CVA21 immunity and the production of CVA21-specific antibodies. After two weeks a boost was given by IP injection of 2 × 10^7^ IFU of CVA21 without the addition of Magic Mouse adjuvant.

Tumors were initiated in 9 week old mice by subcutaneous flank injections of 2 × 10^6^ YUMM 2.1 ICAM-1 cells. Prior to injection, cells were washed once in PBS and once in Hank’s balanced salt solution (HBSS) prior to a final suspension in 100 µL of HBSS. Tumor re-challenge experiments were carried out by preparing and injecting cells in the same manner in the opposing flank.

CVA21 or saline intratumoral injections were administered when tumors reached 100 mm^3^. Mice received a dose of 2 × 10^8^ IFU of virus or equal volume of saline every other day for the first week on days 1, 3, 5, and 8, followed by injections once a week for the next four weeks on days 15, 22, 29, and 36. Tumors were monitored daily and measured three times a week with electronic calipers until the longest diameter of the tumor reached 2 cm or adverse tumor-related complications required animals to be euthanized. Tumor size was calculated using an elliptical estimation:

V = (L × W^2^)/2,

where tumor volume (V) is equal to the length (L) multiplied by the square of the width (W) divided by two.

Tumor responses were classified as progressive disease (PD), partial response (PR), stable disease (SD), or complete response (CR) according to RECIST 1.1 criteria [32]. According to this criteria, PD is determined by a 20% increase in the diameter of target lesion (with no CR, PR, or SD documented before increased disease). PR is classified as a 30% decrease in the longest diameter of the target lesion with recurrence, whereas SD is when neither PR nor PD criteria are met from the smallest diameter since treatment started. CR is classified as the disappearance of the target lesion with no recurrence. Survival curves were generated and analyzed using GraphPad Prism 9.0.1. Survival distributions were compared using a Log-rank (Mantel-Cox) test. *p* values < 0.05 were considered significant (*p* < 0.05 *, < 0.01 **, < 0.001 ***, < 0.0001 ****). All experiments were approved and conducted in accordance with the University of Utah Institutional Animal Care and Use Committee under protocol number 20-07006.

### 2.8. Cytokine and Chemokine Multiplexing

Cytokine/chemokine multiplexing was performed according to the manufacturer’s instructions as described previously [33]. Mouse plasma was collected immediately following euthanasia of animal subjects. Upon sacrifice, 200 µL of blood was collected into tubes coated with 10 µL of 0.25 mM EDTA following cardiac puncture. Blood samples were cleared at 1600× *g* for 10 min at 4 °C. Plasma was then transferred to clean tubes and flash frozen in a methanol-dry ice bath. All samples were stored at −80 °C. Mouse samples were run using a ProcartaPlex Multiplex Immunoassay kit (Invitrogen; EPXR360-26092-901). Plasma samples were thawed at 4 °C with agitation, vortexed, and cleared at 1000× *g* for 10 min at 4 °C. Then, 25 µL of each sample was then added to a 96-well assay plate and incubated with antibody-conjugated magnetic multiplexing beads for 2 h at RT on a microplate shaker at 500 rpm. Following a wash step using a magnetic plate washer, captured analytes were then probed under similar conditions with biotin-labeled detection antibodies for 30 min and washed prior to treatment with Strepdavidin-PE for another 30 min. Following the final wash steps, the beads were re-suspended in reading buffer prior to running the plate on the Luminex MAGPIX platform. Analyte differences between samples were compared using an unpaired, two-tailed t-test with GraphPad Prism software version 9.0.1. *p* values < 0.05 were considered significant (*p* < 0.05 *, < 0.01 **, < 0.001 ***, < 0.0001 ****).

### 2.9. IncuCyte Live Cell Imaging and Analysis

Live cell imaging was performed using the IncuCyte platform. Cells were seeded at a density of 1.4 × 10^4^ cells per well and given time to adhere to the plate for 4 h. Following cell seeding, CVA21 infections were performed for 1 h, after which cells were washed with PBS and incubated at 37 °C. Two pictures of each well were taken every two hours for 72 h to assess cell death over time. Images were collected and compiled into movies using the IncuCyteZoom2016B software.

## 3. Results

### 3.1. CVA21 Infects and Induces Death of Melanoma Cells Expressing ICAM-1

**Figure 1:**
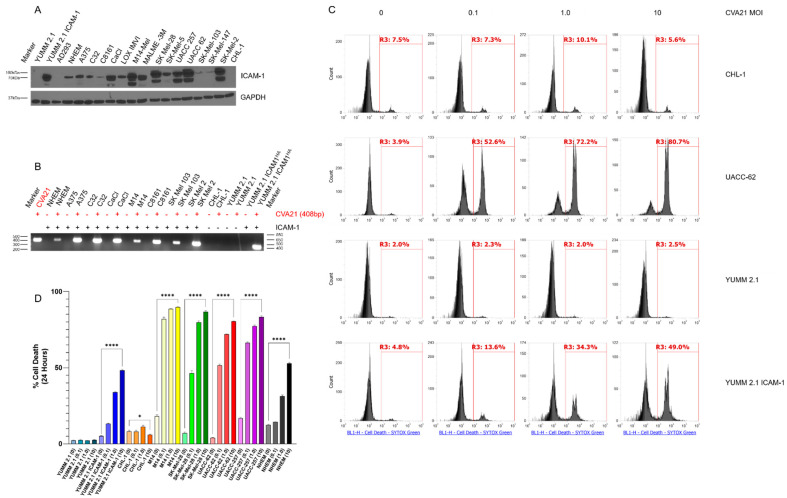
Melanoma cells are susceptible to CVA21 infection and cell death. (**A**) Immunoblot of a panel of melanoma cell lines probing for ICAM-1. GAPDH was used as a loading control. Mouse YUMM 2.1 cells and lentivirally transduced YUMM 2.1 ICAM-1 cells were used as negative and positive controls for ICAM-1, respectively. (**B**) RT-PCR of RNA extracted from a panel of melanoma cell lines infected with CVA21 compared to uninfected parental lines. RT-PCR on purified CVA21 stock was used as a positive control for comparison. The RT-PCR produces an amplicon from the 3′ end of the viral genome in the 3D^pol^. (**C**) Assessment of cell death following CVA21 infection at MOI 0, 0.1, 1.0, and 10 at 24 h by flow cytometry in representative cell lines. SYTOX Green was used to stain dead cells. (**D**) Quantitation of cell death from the flow cytometry assay represented in panel (**C**). Samples were run in triplicate and analyzed using a one-way ANOVA test for statistical differences. Error bars represent the standard deviation between replicates. *p* values < 0.05 were considered significant (*p* < 0.05 *, < 0.0001 ****).

A panel of human melanoma cell lines were assessed for expression of the CVA21 receptor ICAM-1. Cell lysates were collected and immunoblotting was performed following standard procedures (Figure 1A). The Yale University Mouse Melanoma 2.1 (YUMM 2.1) cell line was used as a negative control. An isogenic clone selected from YUMM 2.1 cell lines transduced with a lentiviral vector expressing human ICAM-1 (YUMM 2.1 ICAM-1) was used as a positive control for ICAM-1 expression (the same YUMM 2.1 ICAM-1 clone was used for all experiments throughout these studies). In the cell lines tested, ICAM-1 expression was nearly ubiquitous and found in varying degrees, including in Normal Human Epithelial Melanocytes (NHEM). Notably, CHL-1 cells lacked expression of ICAM-1. From this assessment we conclude that the viral receptor CVA21 exploits for infection is abundantly present in melanoma cells.

In order to test whether melanoma cells could be infected with CVA21, we incubated cells with CVA21 at a multiplicity of infection (MOI) of 1 for 1 h. As CVA21 is incapable of utilizing mouse ICAM-1 [16], YUMM 2.1 cells were used as a negative control for the infection. Following incubation with CVA21, the infectious media was aspirated. Cells were then washed to remove unincorporated virus and incubated in fresh media at 37 °C for 24 h. Media was then removed, cells were washed, and RNA was extracted using TRIzol reagent following the manufacturer’s instructions. RT-PCR was performed and PCR products were run on a 1% agarose gel in order to detect the presence of the viral genome (Figure 1B). RNA from infected cell lines was compared to RNA extracted from uninfected parent cell lines following the same procedures. RT-PCR was also performed on the viral stock directly for comparison. All of the melanoma lines that express ICAM-1 also proved to be susceptible to infection with CVA21. Furthermore, CVA21 infection was not detected in cell lines that do not express ICAM-1, such as in CHL-1 or YUMM 2.1 cells. Nevertheless, when unsusceptible cells were made to ectopically express the receptor (YUMM 2.1 ICAM-1) they became susceptible to infection with the virus.

Once infection of melanoma cells by CVA21 was confirmed, the oncolytic activity of the virus was evaluated. Melanoma cell lines were treated with virus at an MOI of 0, 0.1, 1.0, or 10 for 1 h after which cells were washed and incubated for 24 h. Live and dead cells were then collected for each uninfected or infected line and stained with SYTOX Green vitality stain and analyzed by flow cytometry (Figure 1C). In this assay live cells exclude the dye while dead cells that have compromised membrane integrity are readily stained. Melanoma cells that express ICAM-1 and therefore could be infected showed significantly increased cell death upon infection with CVA21 in a dose-dependent manner, whereas cells that could not be infected did not have an appreciable increase in cell death (Figure 1C,D). Although NHEMs express ICAM-1 and test positive for CVA21 infection they are partially resistant to death (Figure S1), indicative of intact anti-viral defenses. Taken as a whole, these data demonstrate that CVA21 can infect and kill melanoma cells that express ICAM-1.

### 3.2. CVA21 Is Cleared from the Blood of Mice following Systemic or Intratumoral Delivery and Exposure to the Virus Induces a Robust Adaptive Immune Response over Time

**Figure 2:**
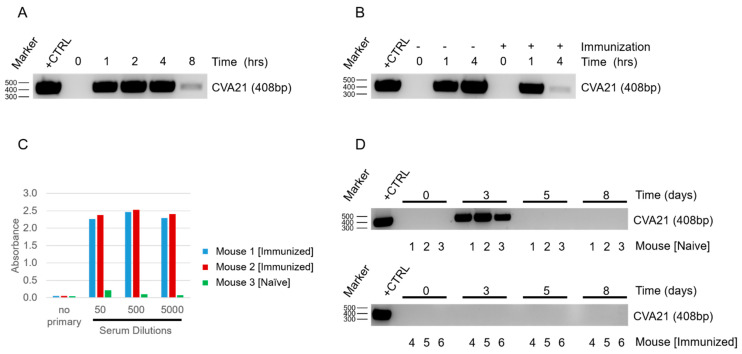
Response to systemic and intratumoral CVA21 exposure in a murine model system. (**A**) RT-PCR time course of CVA21 systemically delivered IP and extracted from the blood of naïve mice. Purified CVA21 stock was used as a positive control for comparison (**A**,**B**,**D**). (**B**) RT-PCR time course comparison of CVA21 systemically delivered IP and extracted from the blood of naïve or immunized mice. (**C**) ELISA test for CVA21 antibodies in the serum of naïve and immunized mice. (**D**) RT-PCR time course of CVA21 in the blood following IT injection of naïve and immunized mice (3 mice/cohort).

As in vitro conditions for the viral infection lack the critical elements of the immune response, viral exposures and treatments were then examined in vivo in murine model systems. In order to test the kinetics of viral clearance from the blood, C57BL6 mice were inoculated with 2 × 10^7^ IFU of CVA21 by intraperitoneal (IP) injection. Blood was drawn prior to inoculation and at 1, 2, 4, and 8 h post-inoculation. RNA was extracted from the blood with TRIzol and quantified using a NanoDrop spectrophotometer (Thermo Fisher). RNA levels were then normalized prior to reverse transcription and amplified using PCR (Figure 2A). CVA21 was detected in the blood following systemic delivery. Viral levels began to drop off at 8 h post-inoculation, demonstrating that naïve mice are capable of clearing CVA21 in a relatively short time frame.

The ability of mice to clear CVA21 from the blood was then compared between naïve and CVA21-immunized cohorts. In order to simulate prior exposure to the virus, mice were inoculated with a mixture of 2 × 10^7^ IFU of CVA21 and Magic Mouse adjuvant by IP injection followed by a boost with CVA21 two weeks later. After waiting an additional two weeks, these immunized mice and a cohort of naïve mice were inoculated with 2 × 10^8^ IFU of CVA21 by IP injection. Blood samples were then collected and assessed for CVA21 as before (Figure 2B). Mice that had previously been exposed to CVA21 cleared the virus even more rapidly than naïve mice, with viral levels dropping within 4 h post-inoculation. The enhanced clearance of CVA21 in immunized cohorts relative to naïve cohorts suggests that the mouse immune system can be engaged to generate a robust anti-viral response.

To determine whether enhanced clearance of the virus corresponded with the development of an adaptive immune response, serum was collected from naïve and immunized mice and tested for antibodies to CVA21 using an ELISA assay (Figure 2C). Serum samples diluted 1:50, 1:500, and 1:5000 were incubated in a 96-well plate coated with CVA21 antigen, after which wells were washed and antibodies detected with anti-mouse IgG secondary antibody conjugated to horseradish peroxidase (HRP). Absorbance was quantified using a plate reader. No signal was detected when no primary antibody was used, and wells treated with serum from naïve mice had no appreciable signal. In contrast, wells treated with serum from immunized mice exhibited strong absorbance indicative of the binding of virus-specific antibodies, thus demonstrating that an adaptive immune response can be elicited upon exposure to CVA21 in this model.

Viral load in the blood was then investigated following intratumoral (IT) delivery of CVA21 in naïve and immunized cohorts. Blood was drawn prior to treatment as a baseline comparison, and tumors were injected with 2 × 10^8^ IFU of CVA21 immediately after. Blood was then drawn prior to repeating IT injections of CVA21 on days 3, 5, and 8 and evaluated by RT-PCR (Figure 2D). Despite demonstrating that CVA21 could be cleared quickly from the animal upon systemic delivery, the virus was detected in the blood of naïve mice 48 h after the first IT injection. The persistence of the virus long after it would have expected to be cleared likely denotes viral replication within the tumor. CVA21 could not be detected in the blood at the later indicated times notwithstanding subsequent tumor injections. Strikingly, CVA21 could not be detected in the blood of immunized mice at any of the time points. Additionally, CVA21 could be detected in distant tumors of naïve but not immunized mice following IT delivery in the primary tumor (Figure S2).

CVA21 does not replicate in normal mouse tissues but can replicate in YUMM 2.1 ICAM-1 tumors. Given that CVA21 does not replicate effectively in normal tissues and that tumors are the primary site of viral replication in humans, this model mirrors the human condition and can evaluate the impact of anti-CVA21 immunity on tumor responses to IT CVA21 therapy.

### 3.3. Pre-Existing Viral Immunity Does Not Markedly Impact Tumor Responses to Treatment with CVA21

**Figure 3:**
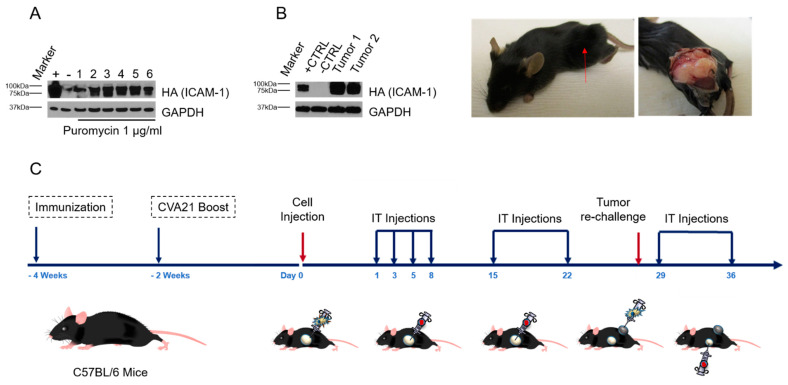
Development of an immune-competent syngeneic tumor model for CVA21 interventions and treatment study design. (**A**) Immunoblot of isogenic clones of YUMM 2.1 cells transduced with a lentivirus expressing ICAM-1 and selected with puromycin. GAPDH was used as a loading control. YUMM 2.1 cells transfected with pDest 12.2 ICAM-1 expression vector and YUMM 2.1 parental cells were used for positive and negative controls, respectively (**A**,**B**). (**B**) Immunoblot and images of YUMM 2.1 ICAM-1 tumors grown in the flank of C57BL6 mice. (**C**) Treatment study design.

In order to test the impact of prior exposure to CVA21 on tumor responses, therapeutic intervention with CVA21 was then investigated. An immune-competent syngeneic tumor model was utilized. The Yale University Mouse Melanoma 2.1 cell line was transduced with a lentivirus containing human ICAM-1 and isogenic clones were selected and tested for ICAM-1 expression by immunoblotting (Figure 3A). One of these YUMM 2.1 ICAM-1 clones was selected and 2 × 10^6^ cells were injected in the right flank of C57BL6 mice. Tumors formed with nearly 100% penetrance and were allowed to grow until tumor burden required sacrifice of the animals. Upon sacrifice, tumors were taken and protein was extracted and probed for ICAM-1 expression by immunoblotting (Figure 3B). ICAM-1 was confirmed in these tumors, indicating that expression is stably maintained throughout the life of the animal.

A treatment study was developed based on this mouse model (Figure 3C). For the study, immunized cohorts were again generated and at 9 weeks, sex and age-matched naïve and immunized mice were injected subcutaneously with 2 × 10^6^ YUMM 2.1 ICAM-1 cells in the right flank and monitored for tumor growth for 7–10 days. Once tumors reached 100 mm^3^, mice were assigned into treatment cohorts to receive either IT saline or IT CVA21. Treatment groups in conjunction with immunization status account for 4 total cohorts in this study. IT treatments were given on days 1, 3, 5, and 8 for the first week of the study, after which IT treatments were given once a week for 4 additional weeks for a total of 8 treatments. A tumor re-challenge was performed by injecting cells in the opposite flank ~30 days after the initial treatment to assess the development of anti-tumor immunity. The study was carried out for 70 days from the initial treatment.

**Figure 4:**
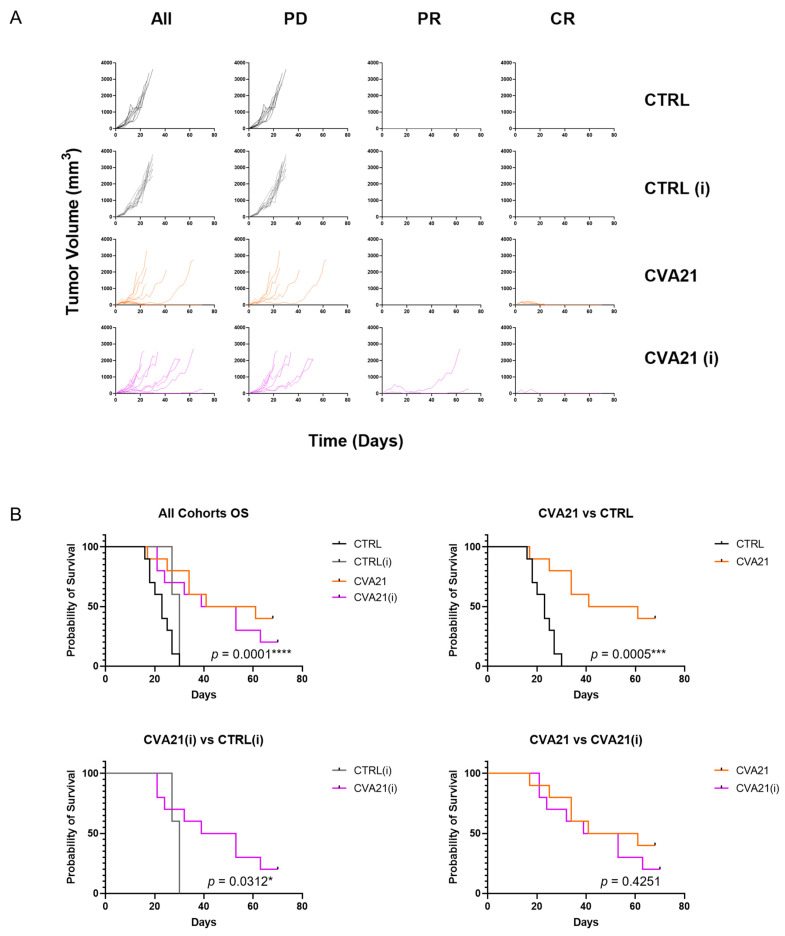
Tumor responses and overall survival in naïve and immunized (i) mice treated with IT saline (CTRL) or CVA21. (**A**) Tumor growth measured in CTRL (*n* = 10), CTRL (i) (*n* = 10), CVA21 (*n* = 10), and CVA21 (i) (*n* = 10) treated mice over 70 days. Responses are classified as progressive disease (PD), partial response (PR), or complete response (CR) according to RECIST 1.1 criteria. (**B**) Survival of CTRL (*n* = 10), CTRL (i) (*n* = 10), CVA21 (*n* = 10), and CVA21 (i) (*n* = 10) over 70 days. Survival distributions were compared using a Log-rank (Mantel-Cox) test. *p* values < 0.05 were considered significant (*p* < 0.05 *, < 0.001 ***, < 0.0001 ****).

Tumor growth was monitored daily and responses were categorized based on RECIST 1.1 criteria into progressive disease (PD), stable disease (SD), partial response (PR), or complete response (CR) [32] (Figure 4A; Table S1). In naïve mice, control tumors were all classified as PD and had a median survival of 23 days. Conversely, CVA21 exhibited impressive tumor regression, with 4 CR out to 70 days (Table S1).

In immunized mice, control tumors were all classified as PD. Although immunized control mice had a slightly increased tumor latency when compared to the corresponding naïve cohort, all mice still required sacrifice due to tumor burden by 30 days, and thus were not ultimately affected by prior exposure to CVA21. When immunized mice were treated with CVA21, 2 PR and 1 CR were achieved (Table S1). This is unexpected considering how quickly the virus is cleared from mice that have had prior exposure, and indicates that CVA21 can be efficacious in spite of a strong immune response.

When comparing the overall survival of each cohort, controls behaved as expected with no mice surviving the duration of the study (Figure 4B). When mice treated with CVA21 were compared, their immunization status appeared to have little effect. These cohorts exhibited statistically similar overall survival (Figure 4B). These results indicate that prior exposure to CVA21 does not drastically affect whether an individual will respond to IT treatment with the virus.

These treatment groups were evaluated for anti-tumor immune responses by assessing the take rate upon tumor re-challenge. Mice that had to be euthanized after re-challenge but prior to expected tumor formation (minimum 7 days) were not scored. None of the control mice survived to the point of re-challenge. Of the 10 mice that survived to meet this criteria (CVA21 *n* = 5; CVA21 (i) *n* = 5), 67% (2/3) that were categorized as PD developed tumors, while 50% (1/2) of PR developed tumors (Table S2). None of the CR (0/5) developed tumors for the duration of the study irrespective of immunization status (Table S2). None of the response groups exhibited the near 100% penetrance of the initial primary tumors, indicating that even in mice that respond poorly to treatment, a baseline level of anti-tumor immunity developed. Some mice that did not completely respond to the treatment were unable to reject the tumor re-challenge. On the other hand, those designated as CR were all able to effectively prevent the outgrowth of a secondary tumor. This observation suggests that the anti-tumor immune response is enhanced in individuals where treatment is effective.

### 3.4. CVA21 Promotes Inflammation and Recruits Inflammatory Cells to the Tumor

Flow cytometry was utilized to profile the landscape of infiltrating immune cells within the tumor to evaluate differences between treatment groups. Naïve and immunized mouse cohorts were established, tumors initiated, and treatment groups assigned as before. Each mouse received an IT injection on day 1 and day 4 according to their assigned treatment group. Mice were euthanized on day 6.

Upon sacrifice, tumors were weighed prior to mechanical dissociation and collagenase (IV) digestion in serum-free RPMI media. Following digestion, supernatants were collected and filtered and red blood cells lysed. Samples were then cleared by centrifugation and re-suspended in RPMI prior to plating and staining with panels for myeloid and lymphoid markers.

**Figure 5:**
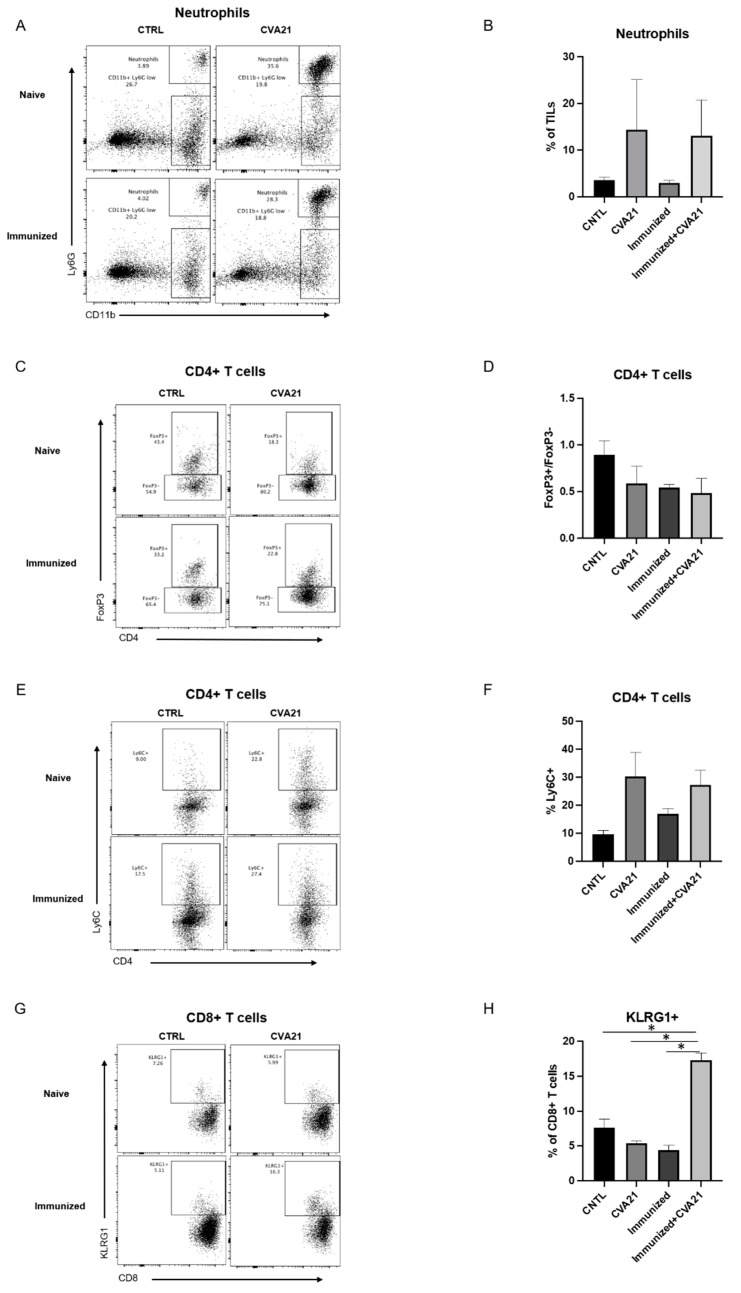
Tumor immune cell profiling by flow cytometry in naïve and immunized mice. Cells were extracted from tumors treated with saline or CVA21 in naïve or immunized (i) mice. Ly6G and CD11b were used as markers of neutrophils (**A**,**B**). FoxP3, Ly6C, and KLRG1 were used to evaluate T cell activation (**C**–**H**). Density plots and quantitation are shown for neutrophils (**A**,**B**), CD4+ T-cells (**C**–**F**), and CD8+ T-cells (**G**,**H**). Data were collected from 3 naïve and 3 immunized mice per treatment group. Error bars represent the standard error of the mean (SEM) between the replicates. *p* values < 0.05 were considered significant (*p* < 0.05 *).

Infiltrating neutrophils were identified using Ly6G and CD11b markers (Ly6G+/CD11b+). In control-treated mice, immunization did not alter the composition of neutrophils within the tumor. When mice were treated with IT CVA21, a comparable influx of neutrophils was observed in both naïve and immunized cohorts, characteristic of an innate antiviral response (Figure 5A,B).

Tumor-infiltrating CD4+ and CD8+ T-cells were also identified and evaluated for different markers of activation. Upon exposure to CVA21, CD4+ T-cells tended to have a decreased Treg to-non-Treg ratio as signified by FoxP3 staining (FoxP3+/FoxP3-) (Figure 5C,D). Additionally, CVA21-treated naïve and immunized mice showed an increase in Ly6C+ CD4+ T cells, indicative of an expansion of short-term effector T cells (Figure 5E,F). There was a significant increase in the percentage of CD8+ T-cells expressing KLRG1, a marker of cytotoxic and proliferative effector cells, in immunized mice treated with CVA21 (Figure 5G,H) [34]. However, KLRG1+ effector CD8+ T cells did not expand upon immunization or treatment with CVA21 alone. Nevertheless, the T-cells found within the tumors of CVA21-treated mice appeared to be more activated. These data show that CVA21 promotes a more pro-inflammatory tumor microenvironment in naïve or immunized subjects.

In addition to infiltrating immune cells, inflammatory cytokines and chemokines were profiled in each cohort using the Luminex Magpix cytokine multiplexing platform. Mice were euthanized at 24, 48, and 72 h following a single dose IT on day 1. Blood was collected by cardiac puncture and plasma extracted into EDTA-coated tubes from naïve and immunized mice. Cytokine multiplexing was performed using magnetic capture beads, with cytokines detected using biotin-conjugated primary antibodies and streptavidin-conjugated PE fluorophore.

Significant increases in IL-23 were observed in immunized cohorts at 24 h (Figure S3). While many of the inflammatory cytokines assayed did not exhibit statistically significant differences, there was a notable trend for cytokine levels to be higher in the plasma of immunized mice. Taken together, the generation of CVA21-specific antibodies, the influx of neutrophils in the tumor, the increase in T-cell activation, and the increase in inflammatory cytokines upon CVA21 exposure portends an immune system that is primed for a response.

## 4. Discussion

CVA21 is an oncolytic virus naturally targeted to human ICAM-1 on the cell surface that can infect and kill melanoma cells (Figure 1) [15,16,17,21]. We have observed that CVA21 replicates well in many human melanoma cell lines, with greater than 50% cell death occurring even at low MOI within 24 h (Video S1). Transduction of YUMM 2.1 mouse melanoma cells with human ICAM-1 rendered them susceptible to viral infection and cell death, however, viral spread appears to occur more slowly in these cells. This may be due to differences in cellular machinery between human and mouse cells that affect their ability to fight off infection. Moreover, the additional mutational burden found in human melanomas corresponds with impaired innate antiviral defenses that render cells vulnerable to viral takeover. This in contrast with our genetically engineered mouse model (GEMM) derived tumors that have much of their normal cellular machinery intact. The same is true of normal human epithelial melanocytes (NHEM), where CVA21 replicates poorly. Despite infecting and killing a high percentage of these cells initially (Figure S1), the virus proves unable to spread and kill NHEMs completely over time. Conversely, we have observed that CVA21 continues to replicate and kill YUMM 2.1 ICAM-1 cells over time. Notably, variance in levels of ICAM-1 expression (Figure 1A) did not determine the infectability of our panel of melanoma cells as confirmed by RT-PCR (Figure 1B). For example, C8161 and SK-MEL-103 cells have low levels of ICAM-1 expression yet exhibit high levels of viral RNA following infection. As ICAM-1 is not found alone on the cell surface, it is not unreasonable to suspect that other receptor molecules, such as decay-accelerating factor (DAF), interact with ICAM-1 to enhance this process [17]. In addition, ICAM-1 levels did not determine how well CVA21 replicated within these cells. What is likely more critical for viral replication is the innate antiviral defenses within these cells. The more impaired these pathways are, the more susceptible the cells are to viral propagation.

Nevertheless, it is evident in melanoma and other cell lines we have tested that CVA21 infection is not detected when ICAM-1 is absent. The lack of cell death further corroborates the absence of productive infection observed in ICAM-1(-) cells treated with the virus (Figure 1C). Collectively, these data demonstrate that CVA21 can effectively kill melanoma cells.

Once we confirmed that CVA21 could infect and kill YUMM 2.1 ICAM-1 tumor cells, we next determined how mice would respond to viral exposure. By delivering the virus systemically and sampling from naïve and infected hosts, we were able to see depletion of CVA21 in the blood within a short time span (Figure 2A). As expected, we observed even more rapid clearance of the virus when mice had been immunized prior to exposure (Figure 2B). We anticipated the difference in viral clearance between naïve and immunized mice to be due to an adaptive response against CVA21. The presence of CVA21-specific antibodies in immunized mice were confirmed by ELISA (Figure 2C). Thus, despite CVA21 being a human pathogen incapable of using murine ICAM-1 for infection, mice still generate an immune response to clear the virus. This finding supports the utility of our model to investigate the impact of natural immunity on CVA21 treatments and tumor responses, which is a concern that needs to be addressed for the clinical application of this virus.

Due to the small size of this picornavirus (~30 nm) it is likely that the antibodies generated against CVA21 are neutralizing [28]. Although naïve mice do not have antibodies against CVA21 initially, we anticipate that repeated IT injections of the virus would be sufficient to promote an adaptive response similar to immunized mice in time. Remarkably, despite the rapid clearance of the virus from the blood of naïve mice upon systemic delivery, CVA21 could be detected in the blood following IT injection 48 h after the first delivery (Figure 2D). Detecting virus days after the initial delivery suggests that CVA21 is replicating within the tumor, as we would expect that even slow shedding of the virus into the blood stream would be quickly cleared and undetectable in the absence of viral amplification. Although we expect CVA21 amplification in the tumors of immunized mice as well, we were not surprised that CVA21 could not be detected in the blood at 48 h or beyond as we know these mice clear virus even more rapidly (Figure 2D). After a few days, CVA21 could no longer be detected in the blood of naïve mice despite additional treatments of CVA21, much like the immunized mice. This is likely due to the development of anti-CVA21 immunity. This demonstrates that mice are capable of clearing the virus and that the generation of adaptive immunity makes this response more robust. 

In our mouse model we observed a high tumor take rate (90–100%) that was not compromised by immunization with CVA21. Additionally we saw strong responses to intratumoral CVA21 (Figure 4). Injections of saline argue that mere irritation of the tumor and inflammation from a needle stick is not sufficient to drive tumor regression. As anticipated, immunization did not affect tumor responses in control treated cohorts when compared to their naïve counterparts. Likewise, CVA21 had similar response rates in naïve or immunized hosts. This is surprising considering how stark the clearance of CVA21 is in immunized mice. Moreover, the difference in overall survival between naïve and immunized cohorts treated with CVA21 was not statistically significant (Figure 4B). These observations suggest that immunity to CVA21 does not portend worse tumor responses when the virus is delivered directly to the tumor. Therefore, the presence of CVA21-targeted antibodies may not be necessary to include as an exclusion criterion for patients receiving IT CVA21 therapy, as was done in the phase II clinical trials [26]. Whether antiviral immunity affects tumor responses to intravenously delivered CVA21 remains to be seen. 

We anticipate that this model will be useful in determining the effects of anti-viral immunity on anti-tumor immunity in relation to other picornaviruses that exploit ICAM-1 for infection. As the effects of anti-viral immunity on anti-tumor immunity are expected to vary greatly depending on the oncolytic agent and tumor in question, we believe there is value in following the principles of this model system to assess the impact of immunity on other investigational OV therapies in appropriate tumor models. We postulate that this model will also be useful in investigating the potential for multivalent treatment strategies in overcoming the negative effects of immunity on OV therapies.

Similarities in responses between naïve and immunized mice treated with CVA21 led us to investigate the mechanism of tumor clearance. We suspect that, outside of oncolysis and viral replication, the immune compartment plays a critical role in driving tumor regression. Our studies found that infection with CVA21 drove an influx of neutrophils to the tumor, as would be expected during the early stages of an innate immune response (Figure 5). Upon exposure to CVA21, there was a trending decrease in regulatory T-cells (FoxP3+/FoxP3-) that typically impede tumor responses. Conversely, CD4+ and CD8+ T-cells were activated within the tumor (Figure 5). In the tumors of CVA21-treated naïve and immunized mice, there appeared to be a similar activation of short-term effector CD4+ T-cells (Ly6C+). However, there was a significant increase in CD8+ effector T-cells (KLRG1+) in mice that were both immunized and treated with CVA21, possibly signifying the development of virus-specific CD8+ effector T-cells. Despite the increase in KLRG1+ CD8+ T-cells, similar tumor responses were observed between CVA21-treated naïve or immunized cohorts. This discrepancy is further evidence to suggest that antiviral immunity does not preclude antitumor immunity.

We know that CVA21 replicates within tumors and an anti-viral response is generated that is capable of clearing CVA21 (Figure 2). Nevertheless, CVA21-treated tumors have a high response rate (Figure 4). In conjunction with increased activation of the immune system upon exposure to the virus, we postulate that CVA21 infection also leads to an anti-tumor response as antigen presenting cells uptake tumor antigens in the process of responding to the viral infection. A strong bias for female responses in this model points to differences in male and female antigens as the driving force for tumor responses (Figure S4). It is possible that the antigens of the male-derived tumor cells is what triggers a more robust female response. In the case of a male mouse, CVA21 replicates within the tumor cells driving an antiviral response but fails to promote robust tumor regression due to the lack of foreign antigens. Conversely, in a female mouse CVA21 infects the tumor and replicates as before but the presence of male antigens within these cells are enough to drive an anti-tumor response. In a model where more neo-antigens are present this bias would likely be mitigated and responses normalized between the sexes, a consideration which we are currently addressing.

Although many studies have established the importance of T-cells in the anti-tumor response, there are many different innate and adaptive cells that are involved in the inflammatory response. The combined effect of innate and adaptive immune cells is likely vital for driving tumor regression. We aim to determine the critical compartments that are involved in tumor clearance by assessing responses to treatment when single or multiple compartments of the immune response are depleted. It will also be important to determine the extent to which viral replication and oncolysis is responsible for tumor clearance.

## 5. Conclusions

We report that IT CVA21 induces durable tumor regression in a mouse model of melanoma irrespective of prior exposure to the virus. We observe that exposure to CVA21 elicits an immune response that enhances viral clearance but does not dramatically affect whether an individual responds to treatment. From our experimental results we conclude that coxsackievirus-targeted antibodies may not be a critical determinant of patient selection for IT CVA21 interventions. 

## Data Availability

The authors confirm that the data supporting the findings of this study are available within the article [and/or] its Supplementary Materials.

## Supplementary Materials

The following are available online at https://www.mdpi.com/article/10.3390/cancers13174462/s1, Figure S1: Assessment of cell death following CVA21 infection at MOI 0, 0.1, 1.0, and 10 at 24 h by flow cytometry in melanoma cell lines, Figure S2: Assessment of CVA21 in treated primary tumors, peripheral blood, and untreated secondary tumors in naïve and immunized mice, Figure S3: Cytokine profiling in naïve and immunized mouse cohorts, Figure S4: Tumor responses in naïve and immunized (i) female (F) and male (M) mice treated with saline or CVA21, Video S1: Live cell imaging of melanoma cell lines infected with CVA21, Table S1: Numbers of individuals and proportion of responders for each treatment cohort in naïve or immunized (i) mice, Table S2: Assessment of secondary tumor growth in treatment cohorts stratified by primary tumor responses. Complete Western Blots are also available in the separate pdf file.
