# Supplementary material for: Prior Exposure to Coxsackievirus A21 Does Not Mitigate Oncolytic Therapeutic Efficacy
Source: Cancers (Basel). 2021 Sep 4;13(17):4462. doi: 10.3390/cancers13174462 (PMC8431599; doi:10.3390/cancers13174462)
Supplement: Supplementary file 1 [file cancers-13-04462-s001.zip › cancers-1337325 Supplementary.pdf]

Supplementary Material: Prior Exposure to Coxsackievirus A21 Does Not Mitigate Oncolytic Therapeutic Efficacy

William J. Burnett, David M. Burnett, Gennie Parkman, Andrew Ramstead, Nico Contreras, William Gravley, Sheri L. Holmen, Matthew A. Williams and Matthew W. VanBrocklin

Table S1. Numbers of individuals and proportion of responders for each treatment cohort in naïve or immunized (i) mice. Responses are classified as progressive disease (PD), partial response (PR), or complete response (CR) according to RECIST 1.1 criteria.

Treatment	All	PD	PR	CR
CTRL	10	10 (100%)	0	0
CTRL	10	10 (100%)	0	0
CVA21	10	6 (60%)	0	4 (40%)
CVA21 (i)	10	7 (70%)	2 (20%)	1 (10%)

Table S2. Assessment of secondary (2˚) tumor growth in treatment cohorts stratified by primary (1˚) tumor responses. Mice surviving to 30 days CVA21 (n=5), CVA21 (i) (n=5) were injected in the left flank with YUMM 2.1 ICAM-1 and observed for outgrowth of secondary tumors. No CTRL or CTRL (i) mice survived 30 days and have been excluded. Responses are classified as progressive disease (PD), partial response (PR), or complete response (CR) according to RECIST 1.1 criteria.

1˚ Tumor Response	# 2˚ Tumors Developed/ # Mice Injected	% Of Mice that Developed 2˚ Tumors
PD	2/3	67%
PR	1/2	50%
CR	0/5	0%

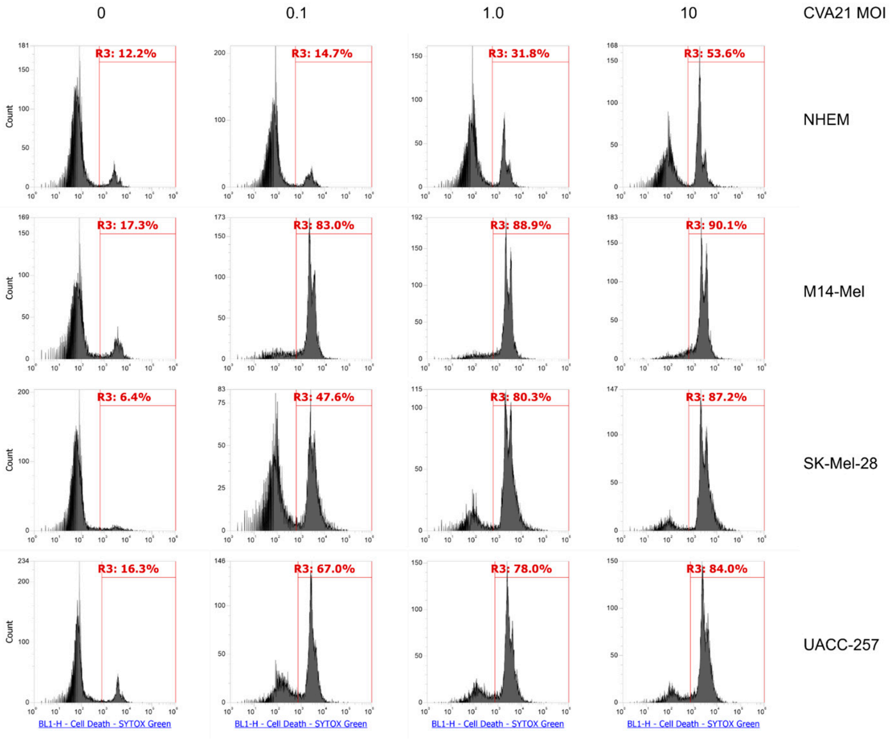

Figure S1. Assessment of cell death following CVA21 infection at MOI 0, 0.1, 1.0, and 10 at 24 hours by flow cytometry in melanoma cell lines. SYTOX Green was used to stain dead cells.

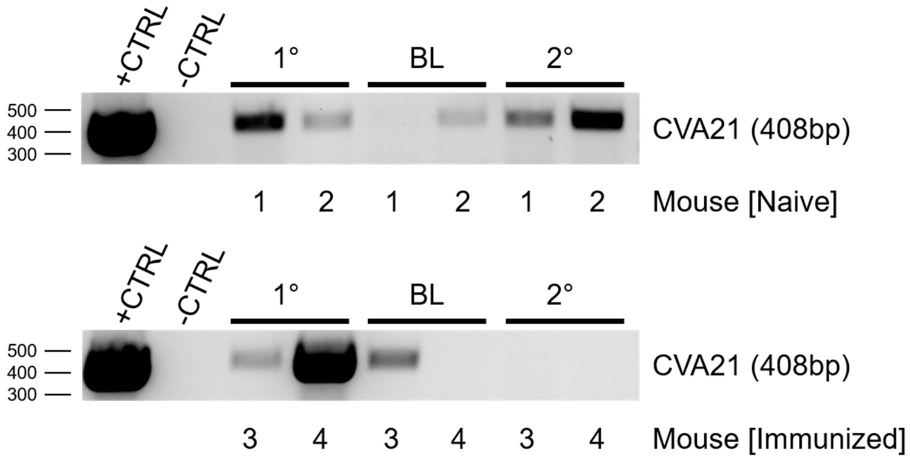

Figure S2. Assessment of CVA21 in treated primary tumors, peripheral blood, and untreated secondary tumors in naïve and immunized mice. RT-PCR of RNA extracted from the primary tumor (1°), peripheral blood (BL), and secondary tumor (2°) of naïve and immunized mice (2 mice/cohort) following IT injection of the primary tumor with CVA21 on days 1 and 3. Mice were euthanized and samples collected on day 5. Purified CVA21 stock was used as a positive control and blood from an untreated naïve mouse was used as a negative control for comparison. .

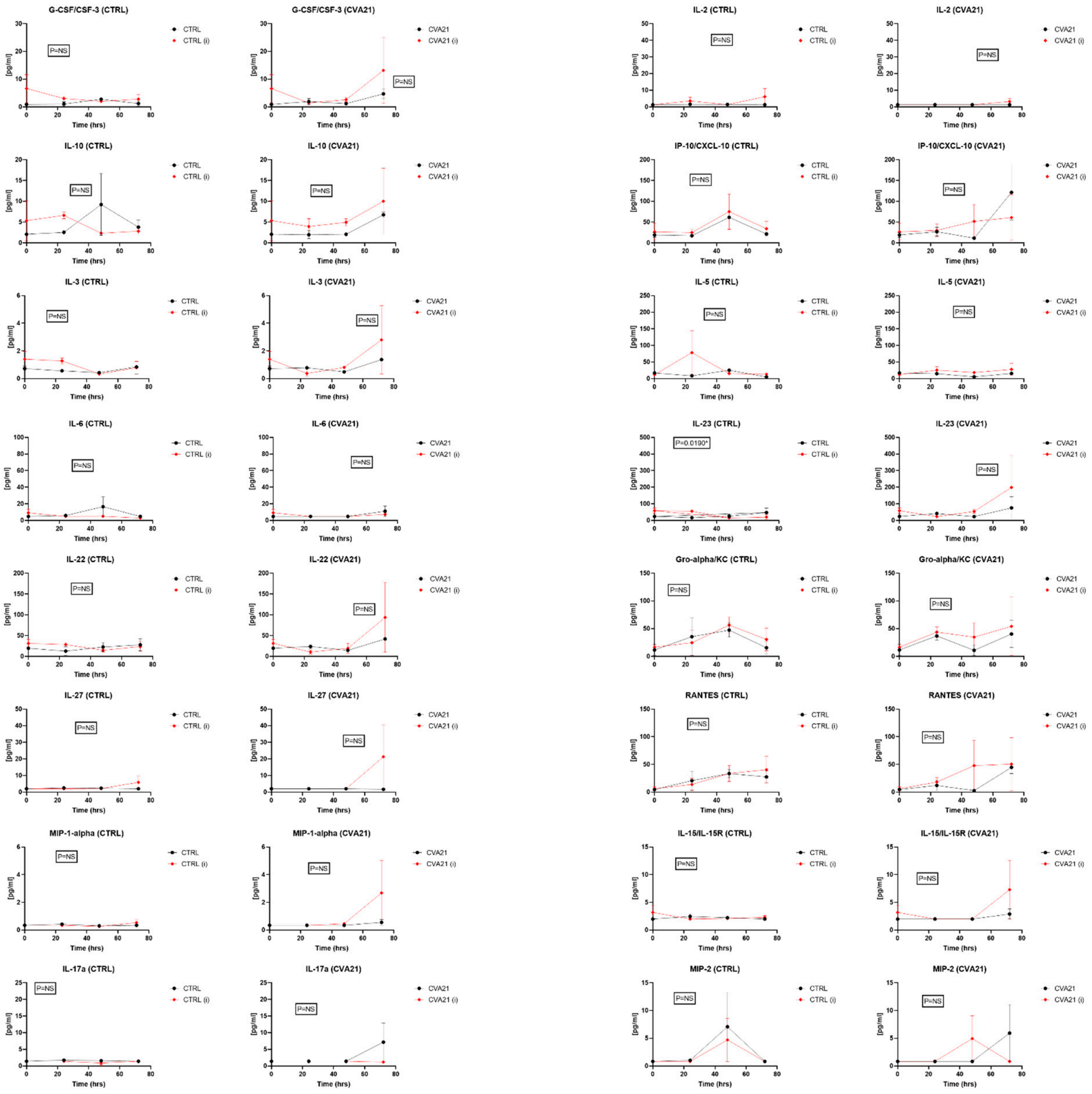

Figure S3. Cytokine profiling in naïve and immunized mouse cohorts. Plasma sampled from mice treated with saline or CVA21 at 24, 48, and 72 hours. Data was collected from 2 naïve and 2 immunized mice per time point. Error bars represent the standard error of the mean (SEM) between the replicates. *P* values <.05 were considered significant (*P:* NS-not significant, <.05*, <.01**, <.001***, <.0001****).

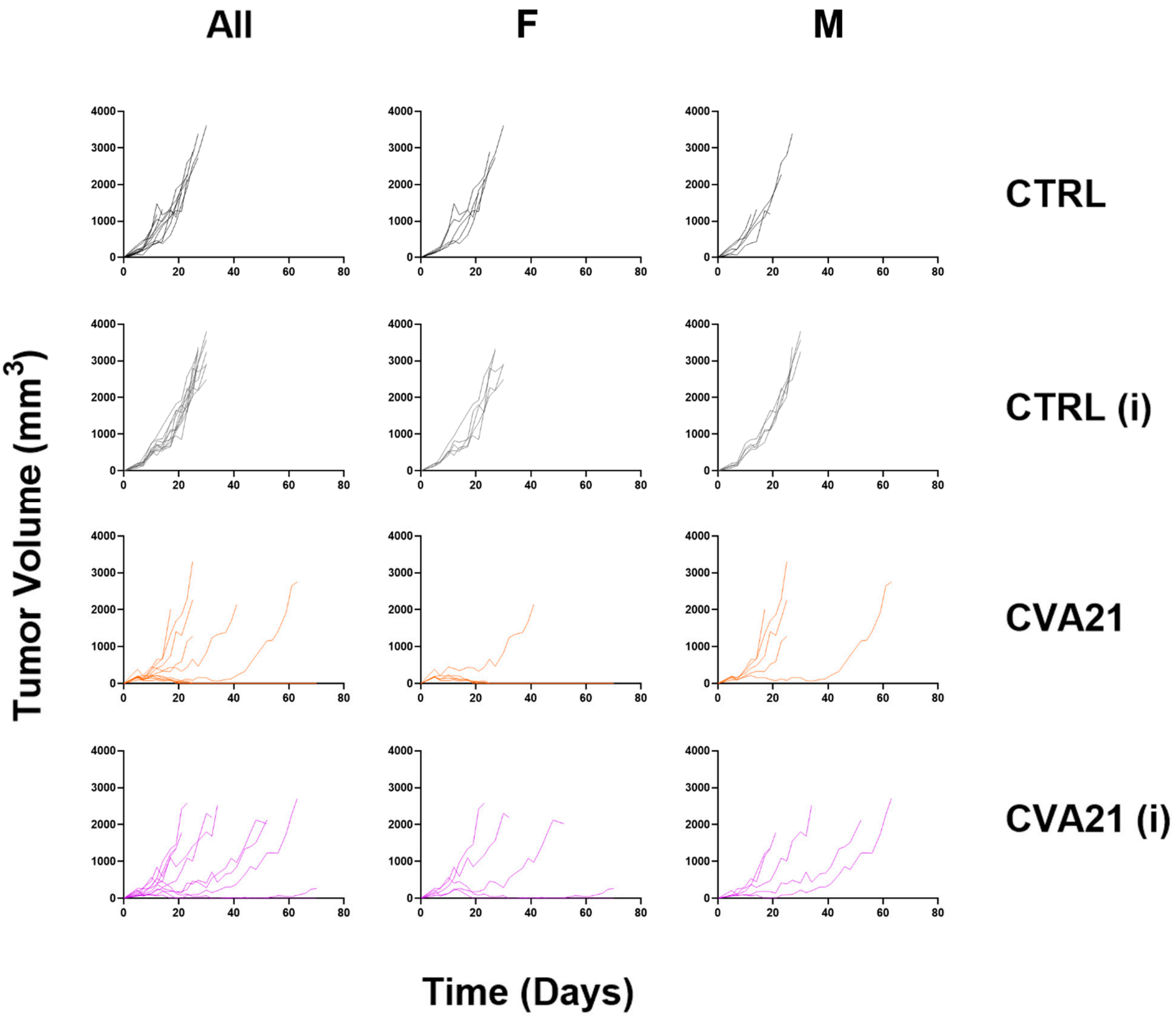

Figure S4. Tumor responses in naïve and immunized (i) female (F) and male (M) mice treated with saline or CVA21. Tumor growth measured in CTRL (n=10), CTRL(i) (n=10), CVA21 (n=10), and CVA21 (i) (n=10) treated mice over 70 days. Responses are classified as progressive disease (PD), partial response (PR), or complete response (CR) according to RECIST 1.1 criteria.

Complete Western Blots are available in the separate PDF file.

Publisher's Note: MDPI stays neutral with regard to jurisdictional claims in published maps and institutional affiliations.

© 2021 by the authors. Licensee MDPI, Basel, Switzerland. This article is an open access article distributed under the terms and conditions of the Creative Commons Attribution (CC BY) license (http://creativecommons.org/licenses/by/4.0/).
