# Supplementary material for: Prior Exposure to Coxsackievirus A21 Does Not Mitigate Oncolytic Therapeutic Efficacy
Source: Cancers (Basel). 2021 Sep 4;13(17):4462. doi: 10.3390/cancers13174462 (PMC8431599; doi:10.3390/cancers13174462)
Supplement: Supplementary file 1 [file cancers-13-04462-s001.zip › Complete Western Blots.pdf]

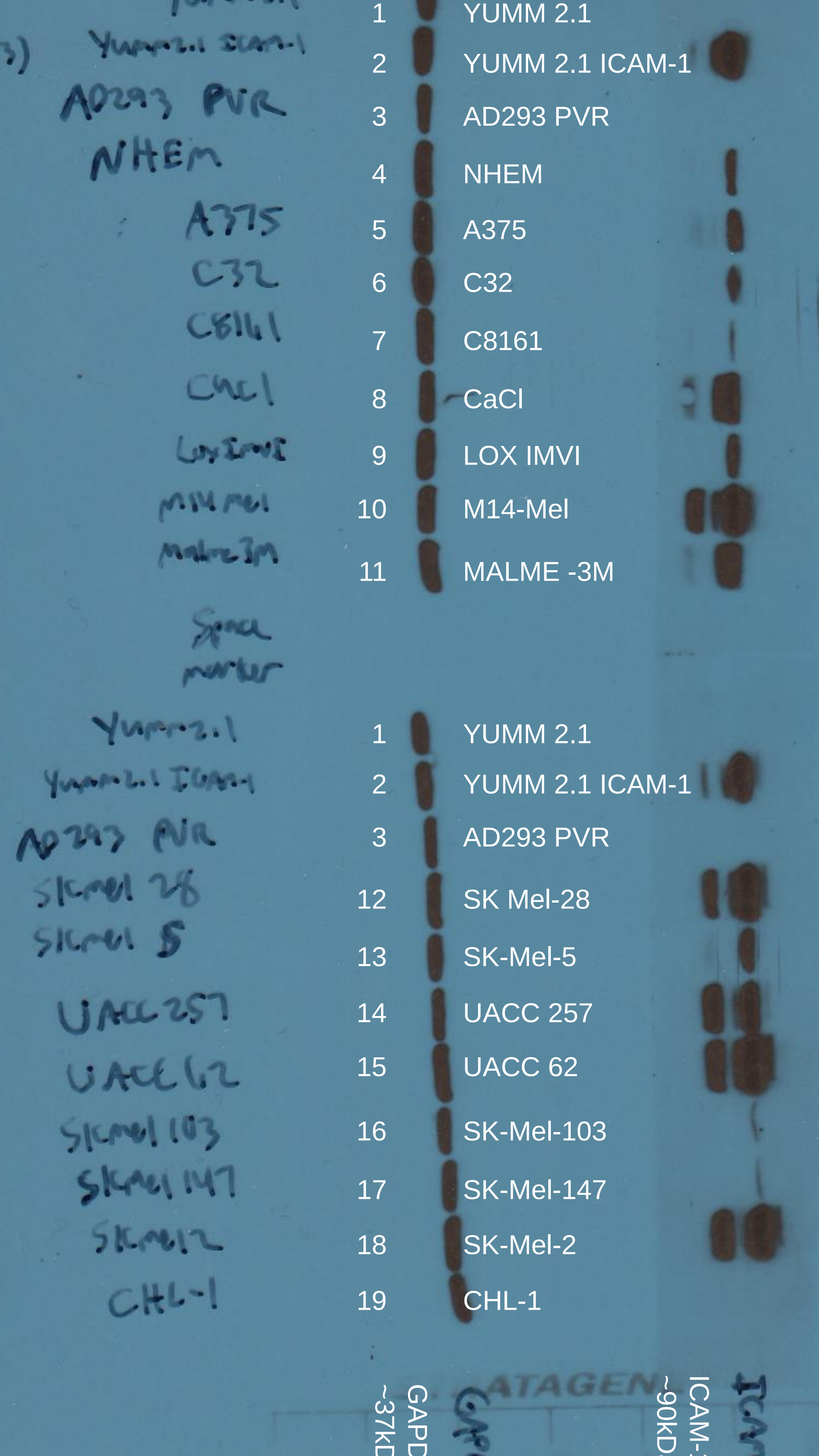
1 YUMM 2.1
2 YUMM 2.1 ICAM-1
3 AD293 PVR
4 NHEM
5 A375
6 C32
7 C8161
8 CaCl
9 LOX IMVI
10 M14-Mel
11 MALME -3M
1 YUMM 2.1
2 YUMM 2.1 ICAM-1
3 AD293 PVR
12 SK Mel-28
13 SK-Mel-5
14 UACC 257
15 UACC 62
16 SK-Mel-103
17 SK-Mel-147
18 SK-Mel-2
19 CHL-1

Figure 1B

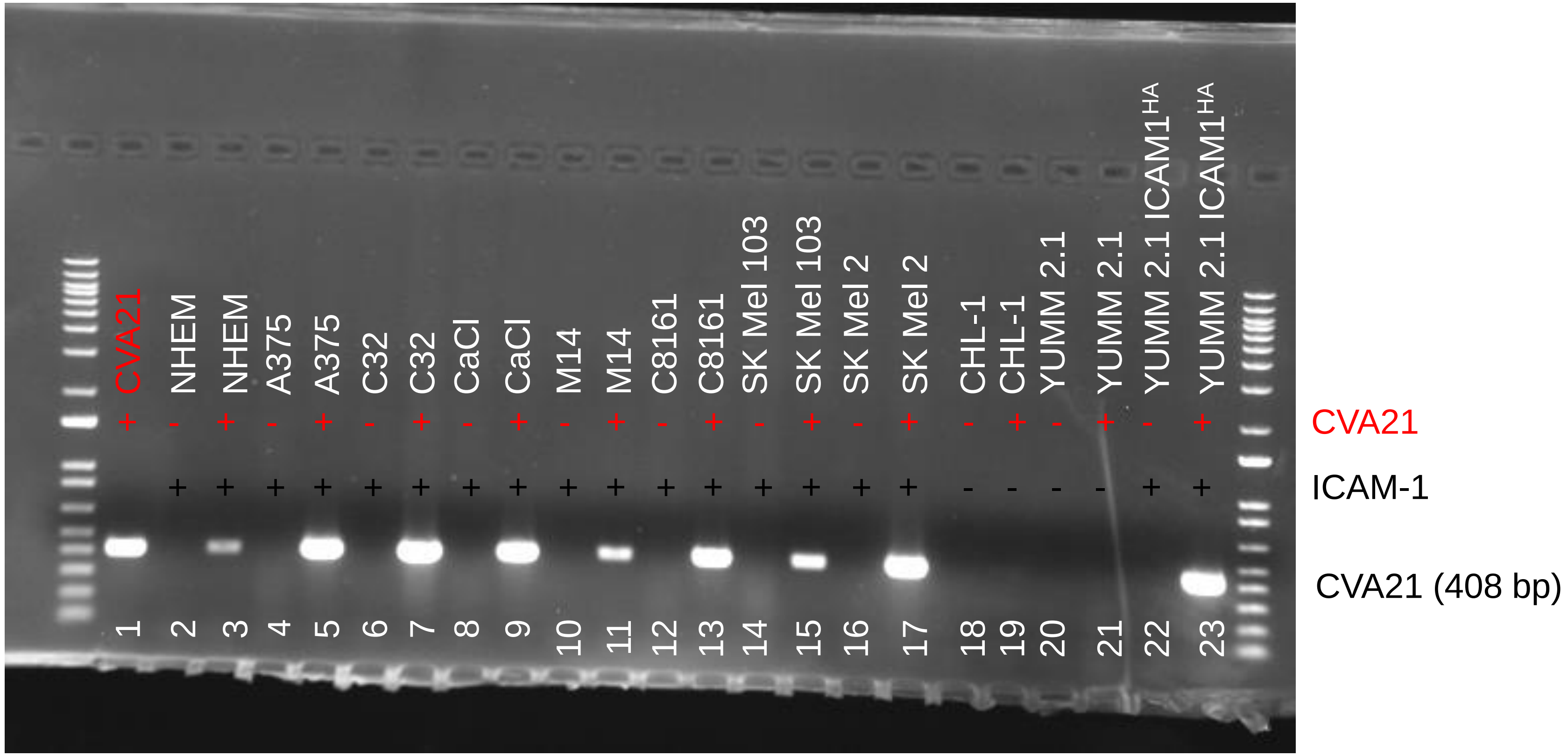

CVA21
NHEM
NHEM
A375
A375
C32
C32
CaCl
CaCl
M14
M14
C8161
C8161
SK Mel 103
SK Mel 103
SK Mel 2
SK Mel 2
CHL-1
CHL-1
YUMM 2.1
YUMM 2.1
YUMM 2.1 ICAM1HA
YUMM 2.1 ICAM1HA
CVA21
+ - + - + - + - + - + - + - + - + - + - + - +
ICAM-1
+ + + + + + + + + + + + + + + + - - - - + +
CVA21 (408 bp)
1 2 3 4 5 6 7 8 9 10 11 12 13 14 15 16 17 18 19 20 21 22 23

Figure 2A

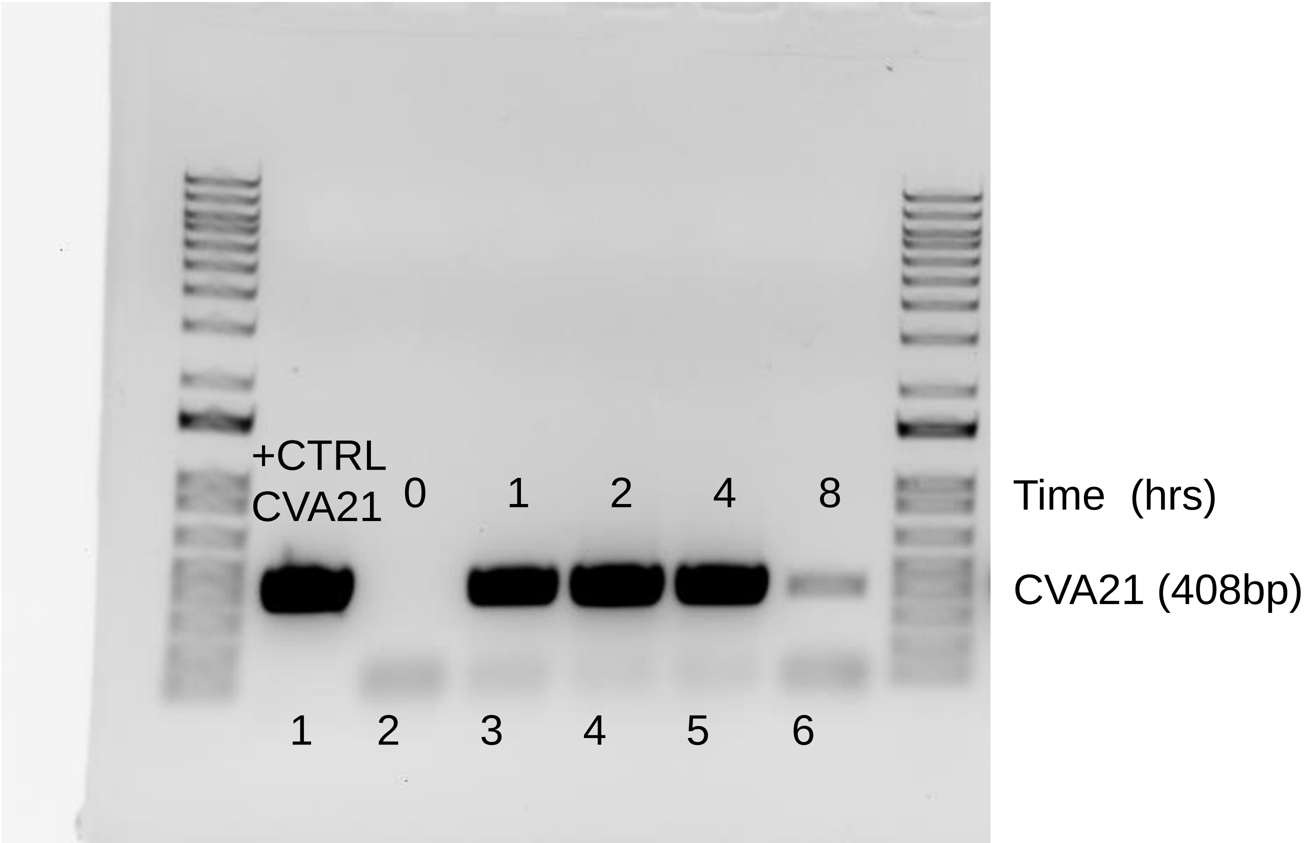

Figure 2B

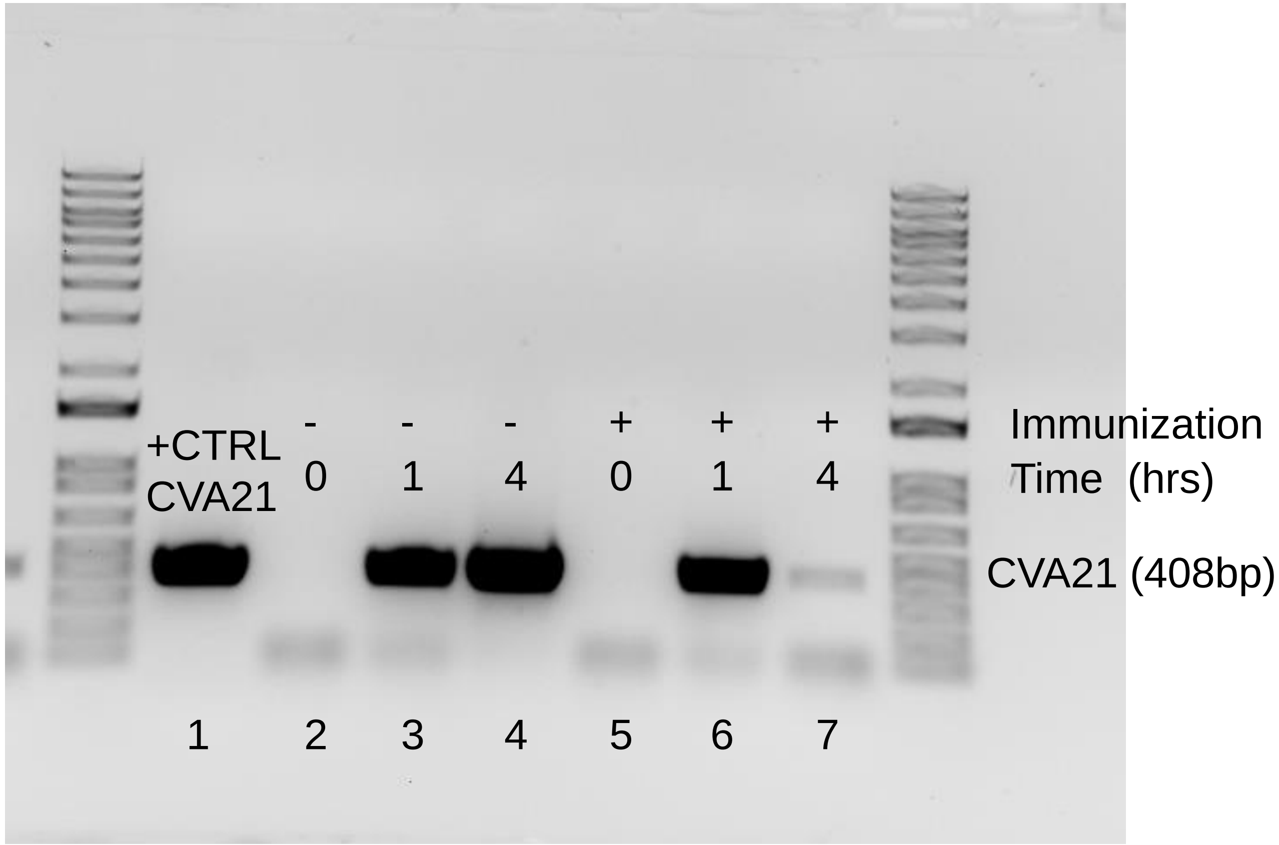
+CTRL
CVA21
- - - + + +
0 1 4 0 1 4
Immunization
Time (hrs)
CVA21 (408bp)
1 2 3 4 5 6 7

Figure 2D

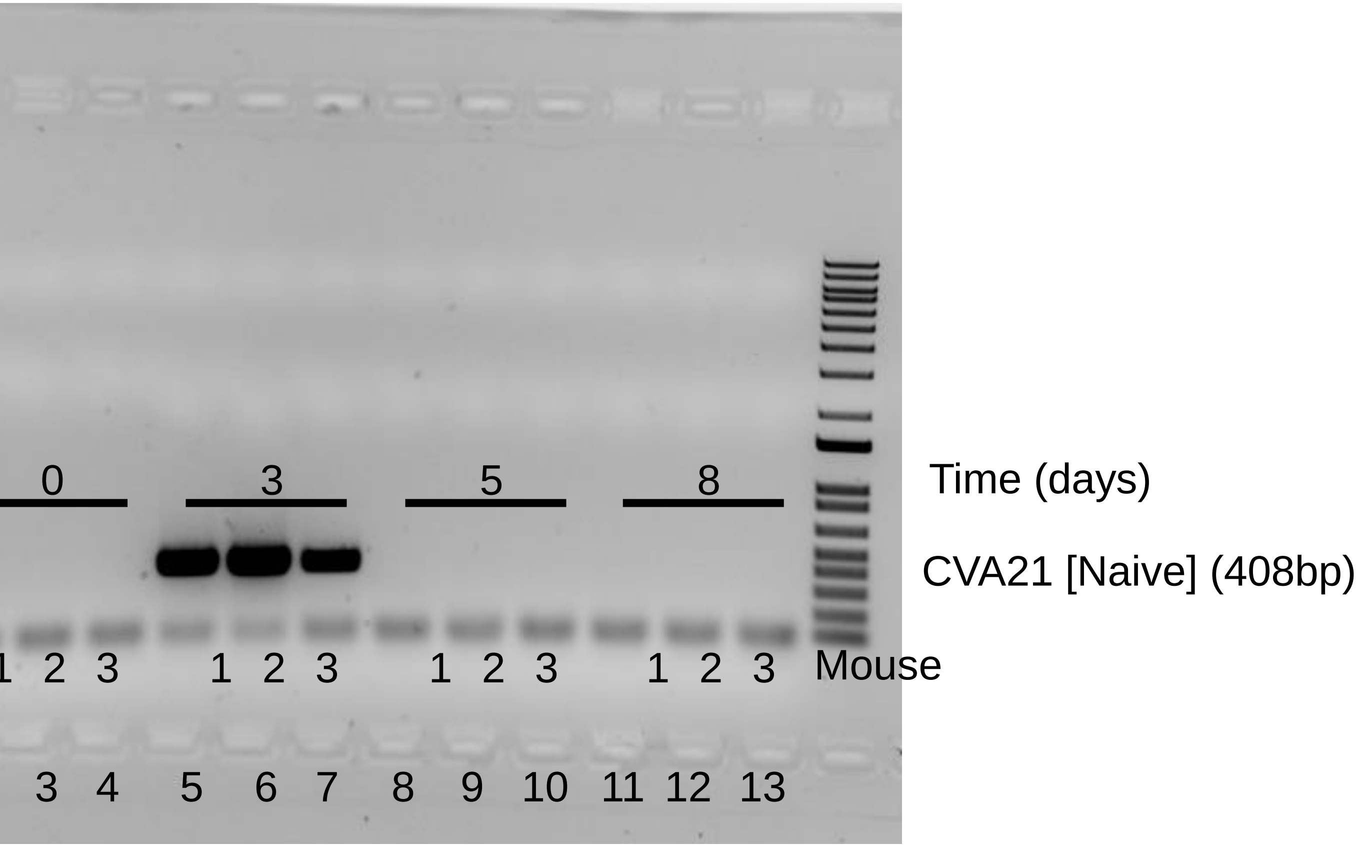
0
3
5
8
Time (days)
CVA21 [Naive] (408bp)
1 2 3 1 2 3 1 2 3 1 2 3 Mouse
3 4 5 6 7 8 9 10 11 12 13

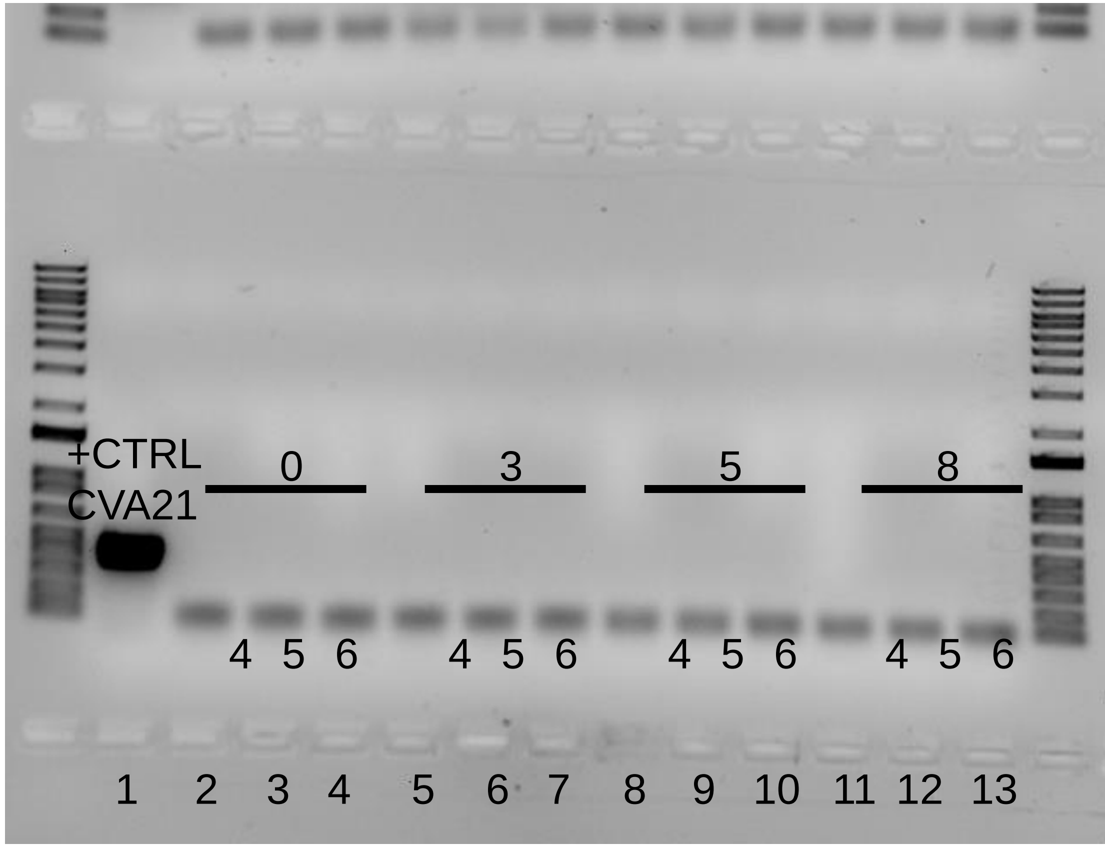
+CTRL
CVA21
0
3
5
8
4 5 6 4 5 6 4 5 6 4 5 6
1 2 3 4 5 6 7 8 9 10 11 12 13

Figure 3A

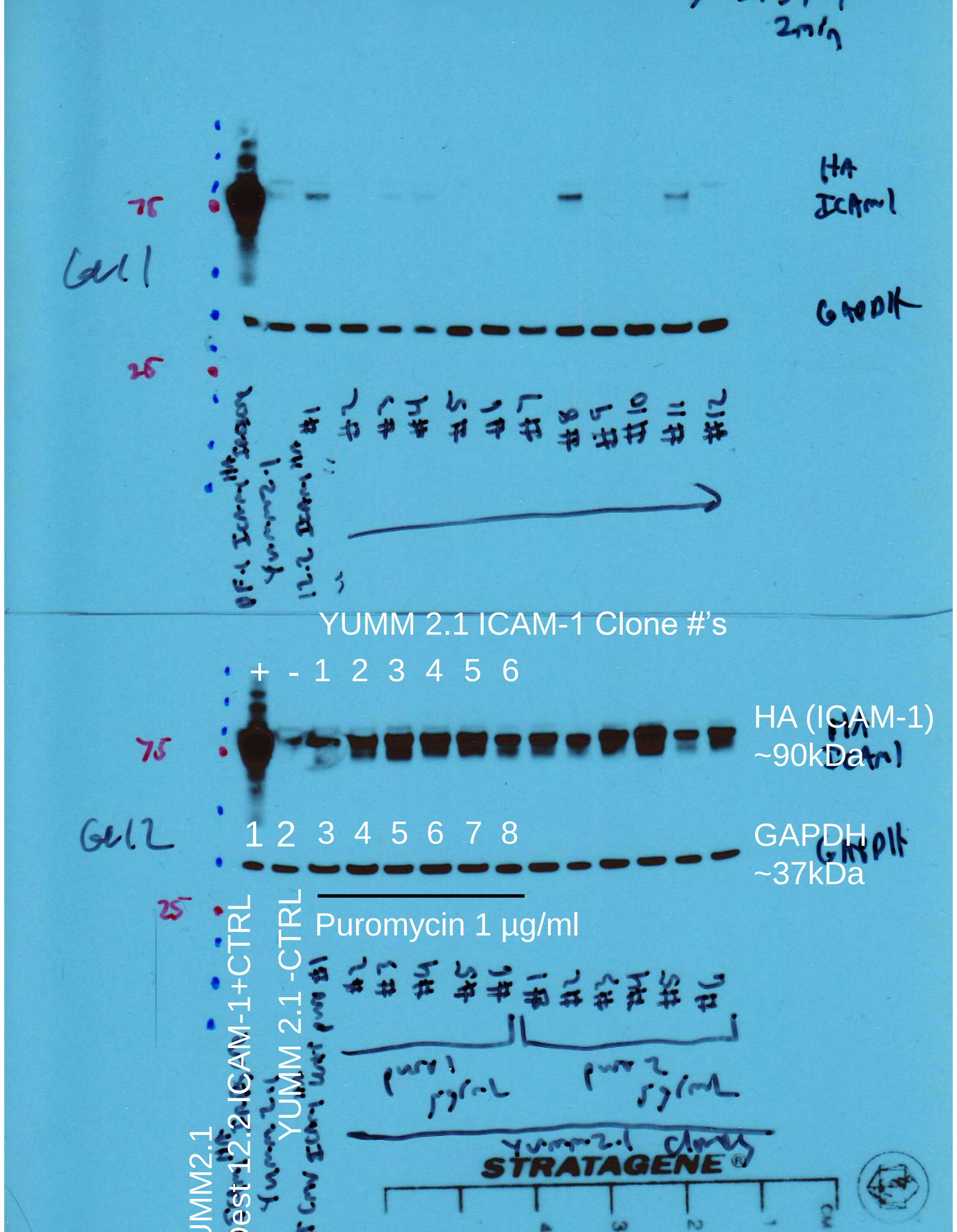
Gel1
YUMM 2.1 ICAM-1 Clone #'s
+ - 1 2 3 4 5 6
HA (ICAM-1)
~90kDa
Gel2
1 2 3 4 5 6 7 8
GAPDH
~37kDa
Puromycin 1 µg/ml
YUMM 2.1 -CTRL
STRATAGENE

Figure 3B

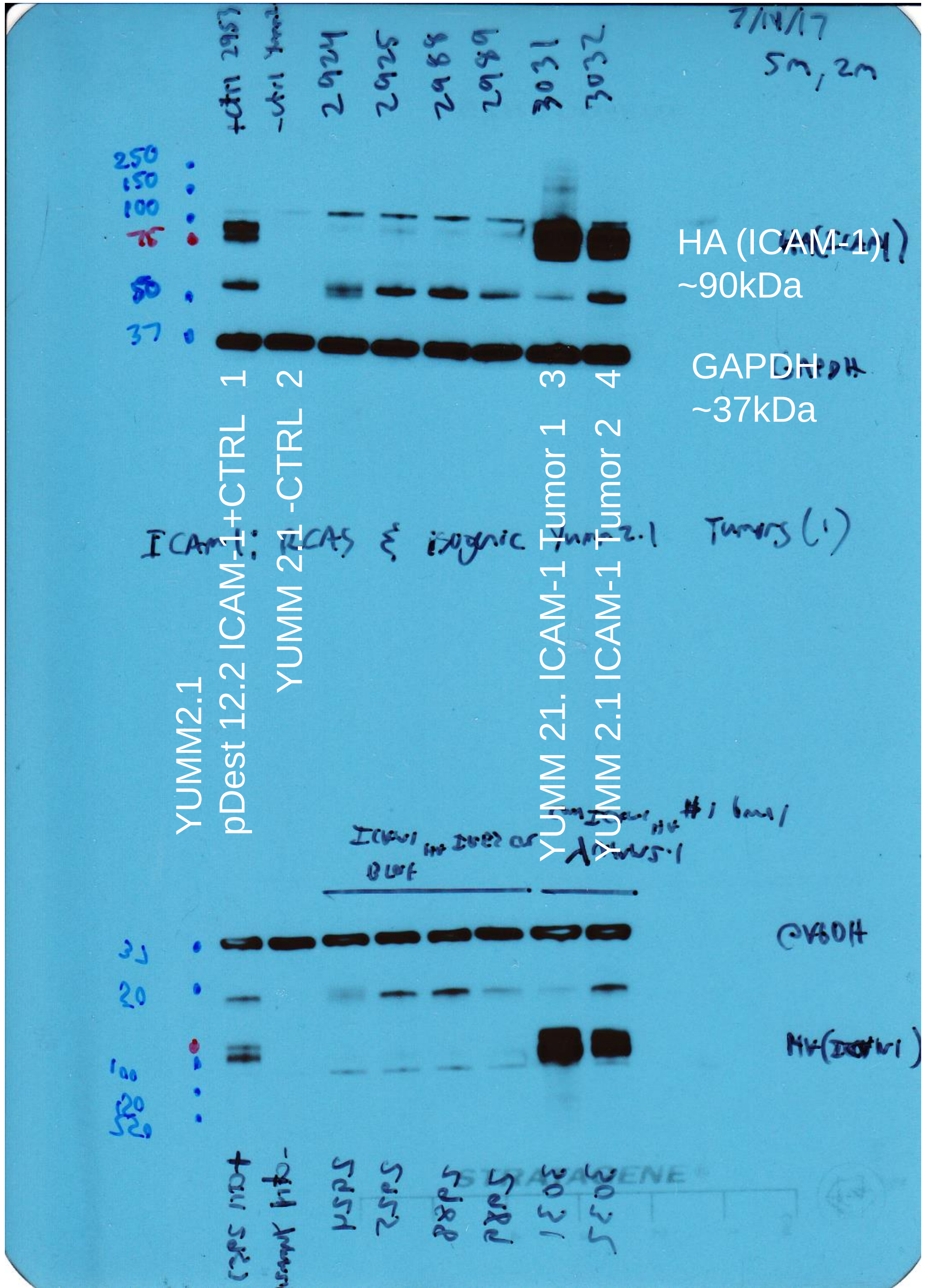

7/14/17
5m, 2m
2924
2925
2988
2989
3031
3032
250
150
100
75
50
37
HA (ICAM-1)
~90kDa
GAPDH
~37kDa
YUMM2.1
pDest 12.2 ICAM-1+CTRL 1
YUMM 21 -CTRL 2
YUMM 21. ICAM-1 Tumor 1 3
YUMM 2.1 ICAM-1 Tumor 2 4
